# Neurological and psychiatric presentations associated with human monkeypox virus infection: A systematic review and meta-analysis

**DOI:** 10.1016/j.eclinm.2022.101644

**Published:** 2022-09-08

**Authors:** James B. Badenoch, Isabella Conti, Emma R. Rengasamy, Cameron J. Watson, Matthew Butler, Zain Hussain, Ben Carter, Alasdair G. Rooney, Michael S. Zandi, Glyn Lewis, Anthony S. David, Catherine F. Houlihan, Ava Easton, Benedict D. Michael, Krutika Kuppalli, Timothy R. Nicholson, Thomas A. Pollak, Jonathan P. Rogers

**Affiliations:** a Barts Health NHS Trust, Charterhouse Square, London EC1M 6BQ, UK; b Preventive Neurology Unit, Wolfson Institute of Preventive Medicine, Queen Mary University of Medicine, London EC1M 6BQ, UK; c Guy's and St Thomas’ NHS Foundation Trust, Westminster Bridge Road, London SE1 7EH, UK; d Department of Public Health and Primary Care, University of Cambridge, Cambridge CB1 8RN, UK; e Institute of Psychiatry, Psychology and Neuroscience, King's College London, WC2R 2LS, UK; f South London and Maudsley NHS Foundation Trust, London BR3 3BX, UK; g Neuropsychiatry Research and Education Group, Institute of Psychiatry, Psychology and Neuroscience, King's College London, WC2R 2LS, UK; h Edinburgh Medical School, University of Edinburgh, Edinburgh EH16 4SB, UK; i Department of Biostatistics and Health Informatics, King's College London, London WC2R 2LS, UK; j Centre for Clinical Brain Sciences, The University of Edinburgh, Edinburgh EH16 4SB, UK; k UCL Institute of Neurology and National Hospital for Neurology and Neurosurgery, Queen Square, London WC1N 3BG, UK; l Division of Psychiatry, University College London, London, UK; m UCL Institute of Mental Health, Maple House, 149 Tottenham Court Road, London W1T 7NF, UK; n UCL Department of Infection and Immunity, University College London Hospital, London NW1 2BU, UK; o Rare and Imported Pathogens Laboratory (RIPL), UKHSA, Porton Down SP4 0JG, UK; p Encephalitis Society, 32 Castlegate, Malton YO17 7DT, UK; q Clinical Infection Microbiology and Immunology, Institute of Infection, Veterinary, and Zoological Science, University of Liverpool, L69 3BX, UK; r Department of Neurology, The Walton Centre NHS Foundation Trust, Liverpool L9 7LJ, UK; s National Institute for Health Research Health Protection Research Unit in Emerging and Zoonotic Infection, University of Liverpool, L69 3BX, UK; t Emerging Diseases and Zoonoses Unit, Department of Epidemic and Pandemic Preparedness and Prevention, Health Emergencies Programme, World Health Organisation, Geneva, Switzerland; u Department of Psychosis Studies, Institute of Psychiatry, Psychology and Neuroscience, King's College London, De Crespigny Park, London SE5 4AF, UK

**Keywords:** Monkeypox, Neurology, Psychiatry, Neuropsychiatry, Encephalitis, Seizure, MPX, monkeypox

## Abstract

**Background:**

Neuropsychiatric presentations of monkeypox (MPX) infection have not been well characterised, despite evidence of nervous system involvement associated with the related smallpox infection.

**Methods:**

In this pre-registered (PROSPERO ID 336649) systematic review and meta-analysis, we searched MEDLINE, EMBASE, PsycINFO, AMED and the preprint server MedRxiv up to 31/05/2022. Any study design of humans infected with MPX that reported a neurological or psychiatric presentation was included. For eligible symptoms, we calculated a pooled prevalence using an inverse variance approach and corresponding 95% confidence intervals. The degree of variability that could be explained by between-study heterogeneity was assessed using the *I*^2^ statistic. Risk of bias was assessed with the Newcastle Ottawa Scale and the Joanna Briggs Institute quality assessment tool.

**Findings:**

From 1705 unique studies, we extracted data on 19 eligible studies (1512 participants, 1031 with confirmed infection using CDC criteria or PCR testing) most of which were cohort studies and case series with no control groups. Study quality was generally moderate. Three clinical features were eligible for meta-analysis: seizure 2.7% (95% CI 0.7–10.2%, I^2^ 0%), confusion 2.4% (95% CI 1.1–5.2%, I^2^ 0%) and encephalitis 2.0% (95% 0.5–8.2%, I^2^ 55.8%). Other frequently reported symptoms included myalgia, headache and fatigue, where heterogeneity was too high for estimation of pooled prevalences, possibly as a result of differences in viral clades and study methodology.

**Interpretation:**

There is preliminary evidence for a range of neuropsychiatric presentations including severe neurological complications (encephalitis and seizure) and nonspecific neurological features (confusion, headache and myalgia). There is less evidence regarding the psychiatric presentations or sequelae of MPX. This may warrant surveillance within the current MPX outbreak, with prospective longitudinal studies evaluating the mid- to long-term sequelae of the virus. Robust methods to evaluate the potential causality of MPX with these clinical features are required. More evidence is necessary to explain heterogeneity in prevalence estimates.

**Funding:**

UKRI/MRC (MR/V03605X/1), MRC-CSF (MR/V007181/1), MRC/AMED (MR/T028750/1) and the Wellcome Trust (102186/B/13/Z) and (102186/B/13/Z) and UCLH BRC.

**Research in context**

**Evidence before this study**

Dermatological manifestations of monkeypox (MPX) infection are well characterised; however, possible neuropsychiatric effects have not been systematically evaluated. There is evidence of nervous system involvement associated with two related *Orthopoxviruses*, in the case of smallpox infection (with the variola virus) and smallpox vaccination (which contains live vaccinia virus). Neuropsychiatric symptoms and complications can be highly disabling and have a detrimental effect on quality of life; consequently, potential nervous system associations of MPX infection are important to recognise. MEDLINE, EMBASE, PsycINFO, AMED and the preprint server MedRxiv were searched up to 31/05/2022 for terms relating to MPX infection and neuropsychiatric features. Any study design was included if it provided data on humans infected with MPX and reported on the presence or absence of neurological or psychiatric clinical features. The majority of studies were of medium quality with significant clinical and statistical heterogeneity.

**Added value of this study**

This systematic review and meta-analysis demonstrates preliminary evidence for a range of neurological and psychiatric presentations of MPX infection. Based on a small number of studies examining this topic, encephalitis, confusion and seizure are present in small (<3%) but non-negligible proportions of infected individuals. The prevalence of other neuropsychiatric symptoms including headache, myalgia, fatigue, anxiety and depression are less clear. There are also knowledge gaps surrounding putative factors which influence the risk of neurological and psychiatric presentations including overall MPX infection severity and viral clade.

**Implications of all the available evidence**

MPX-related nervous system presentations may warrant surveillance within the current monkeypox outbreak, with prospective longitudinal studies evaluating the mid- to long-term sequelae of the virus. Robust methods to evaluate the potential causality of MPX with these clinical features are required at an individual and epidemiological level.

Alt-text: Unlabelled box

## Introduction

Monkeypox (MPX) is a viral zoonotic disease that belongs to the *Orthopoxvirus* genus of the *Poxviridae* family. MPX was first identified in 1958 in monkeys and rodents in a Danish laboratory, and human cases were first identified in the Democratic Republic of Congo in 1970.^1^^,^^2^ MPX virus has historically been classified in two distinct genetic clades. The Central African (or Congo Basin) clade has been described to be more virulent with a case fatality ratio (CFR) ranging from 1% to 10% and the West African (WA) clade, less so, with a mortality of < 3%. The WA clade has been identified as the causal agent of the current outbreak.^3^ Sporadic outbreaks have occurred outside of its ecological niche, including in the USA in 2003 and the UK in 2018.^4^^,^^5^ Since May 13^th^ 2022 a sharp increase in cases, predominantly in the USA and Europe, has brought widened attention to this neglected infectious disease. Concern has arisen due to a high rate of human-to-human transmission and there are current efforts to understand what is driving this transmission.^6^ Negligible global levels of immunity to the smallpox virus and its vaccine is a potential factor because smallpox immunity may provide protection against MPX infection.^7^

While dermatological manifestations in the form of a typically evolving skin rash in patients with MPX are well documented and characterised, other sequelae such as possible neuropsychiatric effects of MPX have yet to be systematically synthesised. Analogous data from smallpox infection and vaccination with vaccinia (a related *Orthopoxvirus*) indicate that neurological and psychiatric features may be significant. Encephalopathy is a common feature of the clinical presentation of smallpox^8^ and, whilst rare, cases of encephalitis, seizures and stroke have been described following both smallpox infection and vaccination.^9^^,^^10^ Encephalitis is estimated to occur in 1 in 500 patients infected with the *Variola major* strain of smallpox and in 1 in 2000 patients infected with the *Variola minor* strain, occurring 6–10 days after infection.^9^ Post-vaccination encephalitis is estimated to occur at a rate of between 2 and 1219 cases per 100,000 vaccines^11^ with higher rates thought to be associated with use of more neurotropic vaccinia strains,^10^ providing *prima facie* support for the relevance of *Orthopoxvirus* biology in the aetiopathogenesis of these sequelae.

In this systematic review and meta-analysis we aimed to (1) summarise the prevalences of neurological and psychiatric presentations of human MPX infection and (2) describe the spectrum of such presentations.

## Methods

This systematic review and meta-analysis was pre-registered on PROSPERO (ID 336649). It is reported according to PRISMA guidelines (checklist is included in Supplementary Table 1).

### Eligibility criteria

Included study types were clinical trials, cohort studies, case-control studies, cross-sectional studies, case series and case reports. Due to the rapidly evolving nature of the literature, pre-prints were included. Included studies reported the prevalence of at least one neurological or psychiatric clinical feature. There were no exclusion criteria based on language. Included studies reported human participants of any age diagnosed with an MPX infection (made either clinically or on the basis of laboratory testing). There was no restriction based on sample size for inclusion in the narrative synthesis; however for inclusion in the meta-analysis studies had to have a minimum of 10 subjects. This was a compromise between maintaining sufficient statistical power and reflecting the nascency of the literature.

### Searches

Ovid was used to search MEDLINE, EMBASE, PsycINFO and AMED without filters or limits up to 31/05/2022. The overall search strategy was to combine terms indicating MPX infection and terms indicating neurological or psychiatric presentations. Text searches and subject headings were used. The full search strategy is presented in the Supplementary Methods. MedRxiv was searched for preprints published in the previous 12 months. Manual searching of the reference lists of included papers and other relevant systematic reviews was performed to identify additional relevant studies. Authors in the field were contacted in an attempt to identify unpublished data.

Screening of titles and abstracts for each article was conducted independently by three of the authors (JB, IC, CJW) using Rayyan QCRI (http://www.rayyan.ai/). Where there was disagreement, articles were included for reviewing in the next stage. The list of potentially eligible full texts was imported to a spreadsheet, where two authors (JB, IC) independently assessed eligibility by comparing studies against the eligibility criteria. Where there was disagreement on the inclusion of a full text, a third author (JPR) arbitrated.

### Data extraction

Two of the authors (JB, IC) independently extracted data from each study. Where relevant data were unclear or missing, study investigators were contacted by email for clarification. Where there were discrepancies between reviewers, the two reviewers discussed and agreed on a consensus.

Outcomes were defined as any neurological or psychiatric presentations in any humans infected with MPX. Data were sought at the level of summary estimates. The specific neurological and psychiatric presentations on which data were collected were derived *post hoc* from the data available in the included papers. All results that were compatible with an outcome in each study were included. Data were also collected for the following study characteristics: study metadata (title, author, citation), country of study population, data collection period, study population, single- vs multicentre, study design, inclusion criteria, exclusion criteria, number with a suspected MPX infection, number in whom MPX infection was confirmed, method of MPX confirmation, number of cases not hospitalised, number of cases hospitalised, number of cases hospitalised and admitted to intensive care, number of cases female, age (mean, SD, median and IQR) of the cases, ethnicity of cases, whether there was a control group, number in the control group, control group description, control group matching parameters, method of identification of neurological or psychiatric presentations, temporality of neurological or psychiatric presentations, number with each available neurological or psychiatric presentation, investigation results, qualitative data, outcome and mortality.

Where an outcome was mentioned in at least one participant in a study, it was assumed that it was not present in any participants in whom it was not mentioned. Where relevant data were only available in graphical representations, manual graphical methods were used to estimate prevalence figures.^12^

### Outcomes, summary measures, and synthesis of results

Results for each outcome were grouped together for analysis. The effect measures sought were period prevalences over the course of the illness. Each included study was tabulated and presented sequentially, summarising its design, participants, outcomes and neurological or psychiatric presentations.

### Meta-analysis

For the meta-analysis, every neuropsychiatric presentation reported by two or more studies was examined. In certain instances, there was evidence of overlapping populations between studies, potentially affecting prevalence estimates. To manage this, where overlap was suspected (e.g., Nigeria:^13^, ^14^, ^15^, ^16^; USA^4^^,^^17^^,^^18^) the study with the largest population was included in meta-analysis. However, if for a given presentation (e.g., encephalitis) the study with the largest population did not report data for that symptom, the study with the next largest population was chosen for that particular symptom. Forest plots with 95% confidence intervals were generated using the *metafor* package in *R version 4.0.2*.^19^ The proportion of the variability in estimates due to between-study heterogeneity was measured with the *I*² statistic. Meta-analytic estimates of pooled prevalences were generated only for those outcomes where the between-study heterogeneity was sufficiently low to allow an interpretable result, which in this study we defined as an *I*² < 60%. A random effects model was used due to methodological heterogeneity between studies. Where between-study heterogeneity was sufficiently low, generalised linear mixed models were generated for each prevalence outcome^20^^,^^21^ before using the inverse variance method with the Freeman–Tukey double arcsine transformation as a comparative sensitivity analysis.^22^ Where heterogeneity was too large for meta-analytic pooling of results, we examined potential causes of the heterogeneity. We conducted an additional sensitivity analysis where only studies that were methodologically similar were included and where two or more studies evaluated the prevalence of a given presentation. Subgroup analyses were planned to investigate heterogeneity where there were five or more included studies for any particular outcome by the following groups: study design (prospective vs retrospective), illness severity and method of diagnosis (serological vs clinical). The threshold for statistical significance was set to p-values of less than 0.05.

### Risk of bias

Risk of bias was assessed using the Newcastle-Ottawa Scale for cohort studies, case-control studies and cross-sectional studies.^23^, ^55^ Domain-specific categorisations are reported in Table 2 and the aggregated categorisations used were 0–3 (poor), 4–6 (fair) and 7–10 (good). For case reports and case series, the Joanna Briggs Institute quality assessment tool was used.^24^ Two authors (JB, IC) assessed each study independently. Where there were discrepancies, a third author (ER) arbitrated. Results for each study were presented and patterns in scores analysed. The overall certainty of the evidence was determined by a consideration of the heterogeneity and the risk of bias for each outcome.

### Patient and public involvement

Due to the urgency of this review, patients and members of the public were not involved in the design of this study. The Encephalitis Society, the world's largest brain inflammation charity, were consulted during the analysis and writing-up stage for assistance in interpretation of the results, and this is reflected by Dr Easton's co-author status.

### Role of the funding source

The study was funded by UKRI/MRC (MR/V03605X/1), MRC-CSF (MR/V007181/1), MRC/AMED (MR/T028750/1) and the Wellcome Trust (102186/B/13/Z) and (102186/B/13/Z) and UCLH BRC. The funders of the study had no role in study design, data collection, data analysis, data interpretation, or writing of the report.

## Results

**Figure 1:**
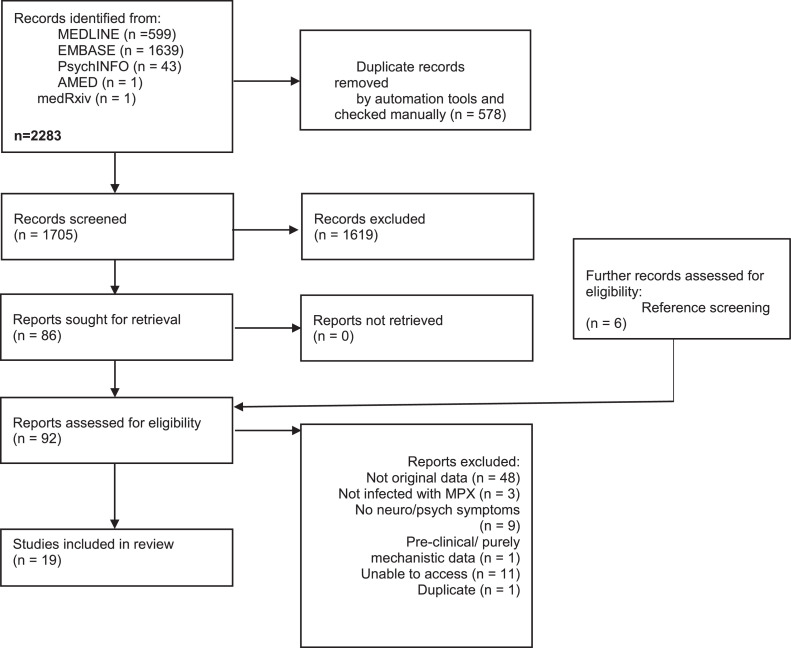
**PRISMA flow diagram**.

The search strategy yielded 2283 studies. After automatic and manual de-duplication, the titles and abstracts of 1705 studies were screened and the full texts of 86 studies were assessed for eligibility. An additional six studies were included from screening references of eligible studies and other relevant systematic reviews. A total of 19 eligible studies were included (see Figure 1 - PRISMA flowchart). Brief reasons for excluding studies are listed in Supplementary Table 2.

### Population and study characteristics

The 19 studies included a total population *n*=1512 individuals (sample size range 1–370; median 21, IQR 81.5) with suspected MPX, *n*=1031 (68.2%) of whom had infection confirmed by PCR or Centers for Disease Control and Prevention (CDC) definitions of confirmed cases. The mean (SD) age reported was 24.2 (19.4) years, based on only 8 studies (*n*=542). Just under half of the population was female (*n*=465, 45.1%). Among studies reporting the setting of MPX treatment (12 studies, *n*=419), most patients were hospitalised (*n*=331, 79.0%). Ethnicity was explicitly reported in only three studies (*n*=54), of which all were from the USA and 46 (85.2%) were White. Most studies originated in the USA (six studies) followed by Nigeria, the Democratic Republic of Congo (five studies each), the Republic of Congo (two studies), and the UK (one study) (Table 1).

**Table 1:** Characteristics of included subjects and studies.

**Characteristics**	
Sample size (median, range, IQR)	21 (1-370, 81.5)
Confirmed Monkeypox (PCR) (n, %)	1031/1512 (68.2)
Age (mean, SD)	24.2 (19.4)
Sex: female (n, %)	465/1031 (45.1)
**Location of MPX treatment** (n, %)	
Hospital	331/419 (79.0)
Community	88/419 (21.0)
Not stated	612
**Country** (k)	
USA	6
Nigeria	5
Democratic Republic of Congo	5
Republic of Congo	2
UK	1
**Year of data collection (latest year, k):**	
2021	1
2019	1
2018	3
2017	1
2016	1
2014	1
2011	1
2010	1
2003	8
1985	1
**Study Design (k)**	
Cohort	12
Cross-sectional	2
Case series	4
Case report	1
Retrospective^a^	7
Prospective^a^	7
Single centre	11
Multicentre	7
Unclear	1
**Newcastle-Ottawa Scale Quality Assessment (k)**^b^	
Low	6
Medium	8
High	0

a only applies to cohort and cross-sectional studies.

b Based on 14 studies (Newcastle-Ottawa Scale used for cohort and cross-sectional studies only).

Most studies (12/19) had a cohort design, two were cross-sectional and the remainder were case series (four) and one case report. Only one study included a comparison group.^25^ There was an equal split of prospective and retrospective cohort and cross-sectional studies.

Risk of bias, assessed using the Newcastle-Ottawa Scale (cohort and cross-sectional studies) and Joanna Briggs Institute Quality Assessment Tool (case series and report), is summarised in Table 2. In the Newcastle-Ottawa Scale, studies lost points on comparability due to a lack of control group in all except one of the included studies. Furthermore, a lack of reported follow-up for the majority of studies also reduced the outcome score on the Newcastle Ottawa Scale.

**Table 2:** Quality assessment scores.

A: Quality Assessment with Newcastle-Ottawa Scale (Cohort and cross-sectional studies)													
	Selection (4)				Comparability (3)			Outcome (3)			Sub-total assessment		
	1	2	3	4	1	2	3	1	2	3	Selection^a^	Comparability^b^	Outcome^c^
**Cohort studies**													
Ogoina et al., 2020^13^	*	-	*	*	-	-	-	*	*	-	Good	Poor	Good
Huhn et al.,2005^25^	*	*	*	-	-	-	-	*	-	-	Good	Poor	Poor
Yinka-Ogunleye et al. 2019^15^	*	*	*	*	-	-	-	*	-	-	Good	Poor	Poor
Boumandouki et al., 2007^29^	*	-	-	-	-	-	-	*	-	-	Poor	Poor	Poor
Akar et al., 2020^16^	*	-	-	-	-	-	-	*	-	-	Poor	Poor	Poor
Croft et al., 2007^28^	-	-	*	*	-	-	-	*	*	*	Fair	Poor	Good
Adler et al., 2022^25^	*	-	*	-	-	-	-	*	-	-	Fair	Poor	Poor
Reed et al., 2004^17^	*	-	*	-	-	-	-	*	-	-	Fair	Poor	Poor
Reynolds., 2006^18^	*	-	*	-	-	-	-	*	-	-	Fair	Poor	Poor
Ježek et al., 1987^30^	*	-	*	-	-	-	-	*	-	-	Fair	Poor	Poor
Kalthan et al., 2016^33^	*	-	*	-	-	-	-	*	-	-	Fair	Poor	Poor
Pittman et al., 2020^32^	*	-	*	-	-	-	-	*	*	-	Fair	Poor	Good
**Cross-sectional studies**													
Ogoina et al., 2019^14^	*	*	*	*	-	-	-	-	*	-	Good	Poor	Poor
Hughes et al., 2021^25^	-	-	*	*	-	-	-	*	*	*	Fair	Poor	Good

B: Quality Assessment with Joanna Briggs Quality Assessment Tool (case series)											
Study	Inclusion criteria^aa^	Measurement of condition^ab^	Identification of condition^ac^	Consecutive inclusions^ad^	Complete inclusion of participants^ae^	Reporting of participant demographics^af^	Reporting of clinical information^ag^	Outcome reporting^ah^	Presenting site(s)/clinic(s) demographics^ai^	Statistical analysis appropriate^aj^	Overall assessment†
Learned et al., 2005^34^	*	*	*	*	*	*	*	*	*	-	Good
Sejvar et al., 2004^26^	*	*	*	-	*	*	*	*	*	-	Good
Reynolds et al., 2006^18^	*	*	-	*	*	*	*	-	*	-	Good
Eseigbe et al., 2021^31^	-	*	*	-	-	-	-	-	*	-	Poor

C: Quality Assessment with Joanna Briggs Quality Assessment Tool (case report)													
Domain	Outcome												
Were patient's demographic characteristics clearly described?	✓												
Was the patient's history clearly described and presented as a timeline?	✓												
Was the current clinical condition of the patient on presentation clearly described?	✓												
Were diagnostic tests or assessment methods and the results clearly described?	✓												
Was the intervention(s) or treatment procedure(s) clearly described?	✓												
Was the post-intervention clinical condition clearly described?	x												
Were adverse events (harms) or unanticipated events identified and described?	✓												
Does the case report provide takeaway lessons?	✓												

Number of asterisks indicates total score for a domain out of the bracketed total in the column heading.

*indicates a domain was met. No studies had statistical analysis, so the domain was not relevant.

a Domain score: 0-1 (Poor), 2 (Fair), 3+ (Good).

b Domain score: 1 (Poor),1 (Fair), 2+ (Good).

c Domain score: 0-1 (Poor), 2+ (Good).

aa Were there clear criteria for inclusion in the case series?

ab Was the condition measured in a standard, reliable way for all participants included in the case series?

ac Were valid methods used for identification of the condition for all participants included in the case series?

ad Did the case series have consecutive inclusion of participants?

ae Did the case series have complete inclusion of participants?

af Was there clear reporting of the demographics of the participants in the study?

ag Was there clear reporting of clinical information of the participants?

ah Were the outcomes or follow up results of cases clearly reported?

ai Was there clear reporting of the presenting site(s)/clinic(s) demographic information?

aj Was statistical analysis appropriate?

† Domain score: 0-3 (Poor), 4-6 (Fair), 7-10 (Good).

Study populations were mostly drawn from national case surveillance projects (e.g., Nigeria:^15^^,^^16^; USA^4^^,^^17^: or cohort studies evaluating the same outbreak of MPX^18^^,^^26^^,^^27^), as shown in Table 3. Other populations were more selective, including a sample of individuals co-infected with Varicella zoster virus^25^ or an evaluation of veterinary workers exposed to an infected prairie dog.^28^ All studies confirmed MPX infection with PCR, except for Boumandouki and colleagues,^29^ and CDC definitions of confirmed cases were followed in most studies. Nine studies confirmed the clade of MPX isolated in infected individuals. Of these, the majority were West African variants, including all six studies in the USA. Two studies reported smallpox vaccination status,^29^^,^^30^ of which the latter found deaths from MPX infection were confined to those not vaccinated for smallpox. Mortality was reported in ten studies and varied between 0-25% in studies with 10 or more individuals.

**Table 3:** Summary of included studies.

Study:	n^a^:	Country:	Clade/ strain:	Date^b^:	Study population:	Neuropsychiatric features:	Other clinical detail:
Ogoina et al. 2020^13^	40	Nigeria	-	09/2017–12/2018	Individuals hospitalised with monkeypox in specific states of Nigeria	Headache 19, myalgia 25, seizure 1, encephalitis 3, photophobia 9, anxiety and depression 11, suicide 1	Those with anxiety and depression required psychological counselling as inpatients
Ogoina et al. 2019^14^	18	Nigeria	-	09/2017–12/2017	Individuals treated at Niger Delta University Teaching Hospital	Headache 12, myalgia 5, pain 5, photophobia 3, suicide 1	Majority expressed fear and anxiety over facing stigma and discrimination from hospital staff
Yinka-Ogunleye et al., 2019^15^	118	Nigeria	West African	09/2017–09/2018	National case surveillance study	Headache 61, myalgia 42, photophobia 27	
Akar et al., 2020^16^	165	Nigeria	-	09/2017–06/2019	Monkeypox cases reported to the Nigeria CDC	Headache 78	
Eseigbe et al., 2021^31^	2	Nigeria	-	2018	First reported nigerian monkeypox cases admitted to Bingham University Teaching Hospital in Jos, Plateau State	Headache 2	
Hughes et al., 2021^25^	134	DRC	-	09/2009–04/2014	Individuals co-infected with VZV and monkeypox, identified through a surveillance program in Tshuapa Province	Headache 99, myalgia 90, fatigue 115	
Pittman et al., 2022^32^	216	DRC	-	03/2007– 08/2011	Patients admitted to General Reference Hospital of Kole the rainforest of the Congo River basin	Headache 49, myalgia 15, dizziness 3, visual deficit 5, confusion 4, fatigue 11	Follow-up assessment at discharge: confusion 1, lethargy/stupor 1
Ježek et al., 1987^30^	209	Zaire/DRC	-	1980–1985	Public health surveillance programme	Encephalitis 1, coma 1	Reported that headache was common but no figure given. Three-year-old unvaccinated girl developed encephalitis and died in a coma on the second day of admission.
Boumandouki et al., 2007^29^	0 (8 unconfirmed)	DRC	-	05/2003–07/2003	Outbreak surveillance study in DRC	Myalgia 2	
Kalthan et al.,2016^33^	12	Central African Republic	-	12/2016– 02/2016	Individuals diagnosed with monkeypox in the district of Bangassou	Headache 2	
Huhn et al., 2005^4^	34	USA	West African	06/2003	Individuals identified through CDC surveillance with monkeypox during 2003 midwest USA outbreak	Headache 23, myalgia 19, seizure 1, confusion 2, encephalitis 1	Six-year old girl who underwent intubation and mechanical ventilation for encephalitis
Croft et al., 2007^28^	19	USA	West African	05/2003–13/2003	Veterinary workers exposed to infected prairie dogs	Headache 13	
Reed et al., 2004^17^	11	USA	West African	05/2003–06/2003	Department of health / CDC outbreak investigation in Wisconsin (all linked to Prairie dog exposure)	Headache 11, myalgia 1	Neurological examinations normal in all patients
Reynolds et al.,2006^18^	37	USA	West African	05/2003–07/2003	Wisconsin outbreak investigation	Headache 32, myalgia 36	
Anderson et al, 2003^27^	1	USA	West African	05/2003–07/2003	Midwest USA outbreak case report	Headache 1, myalgia 1, fatigue 1	No focal neurological signs on admission
Sejvar et al., 2004^26^	3	USA	West African	05/2003	Family cluster in Midwest outbreak	Headache 2, seizure 1, altered mental status 1, delirium/encephalopathy 1, encephalitis 1	Six year old girl with encephalitis: unresponsive, pupillary dilatation, muscle rigidity
Adler et al., 2022^5^	7	UK	West African	08/2018 – 09/2021	Patients admitted to high consequence infectious diseases centres in the UK	Headache 1, pain 1, low mood 3, emotional lability 1	Patient with low mood and emotional lability also had alcohol withdrawal
Learned et al.,2005^34^	3	ROC	-	04/2003–05/2003	Outbreak within a community in Impfondo	Headache 1, irritability 2, distress 4, fatigue 2,	
Reynolds et al.,2013^35^	2	ROC	Congo Basin	04/2010–11/2010	Surveillance study established in in Likouala region	Headache 1, fatigue 1	

a n with confirmed monkeypox.

b time period of data collection. Centre for Disease Control (CDC) Democratic Republic of Congo (DRC) Republic of Congo (ROC)

Neurological and psychiatric presentations varied widely; however, the most frequently reported were headache, myalgia, seizure, confusion, encephalitis and fatigue (Table 3). Neuropsychiatric features were mostly evaluated through case note review in retrospective studies and a mixture of clinical interview and questionnaire in prospective studies. Method of diagnosis of neurological complications, such as encephalitis was variable, with some reports of CSF confirmation,^26^ however, this was not ubiquitous.^13^ The breadth of clinical features assessed in prospective studies was minimal. For example, the only neuropsychiatric presentations assessed in two prospective studies were headache, fatigue and myalgia by Yinka-Ogunleye and colleages^15^ and headache by Croft and colleagues.^28^ Assessment of clinical feature severity, using standardised scales, and chronicity or temporal trajectory of these features was also lacking.

### Prevalence of neurological and psychiatric presentations

**Figure 2:**
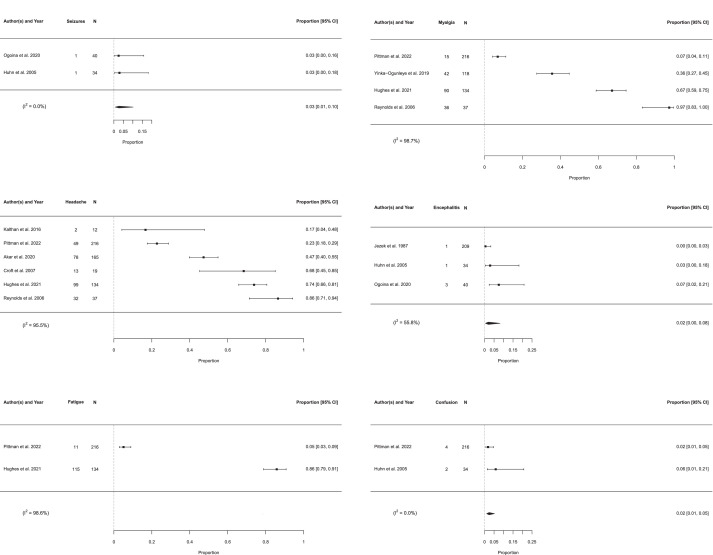
Forest plots for individual neurological and psychiatric presentations.

After exclusion of potentially overlapping populations, six neuropsychiatric presentations were eligible for meta-analysis of prevalence. Forest plots are displayed in Figure 2. Due to high heterogeneity, no pooled prevalences could be calculated for myalgia, headache or fatigue. Pooled prevalences were calculated for seizure (2.7% [0.7–10.2%]), confusion (2.4% [1.1–5.2%]) and encephalitis (2.0% [0.5–8.2%]), as shown in Table 4. Heterogeneity varied between clinical features with *I*^2^ ranging from 0.0% to 55.8% for the outcomes with pooled prevalences and from 95.5% to 98.7% for the outcomes in which estimation of pooled prevalences was not undertaken. Other neuropsychiatric features including depression, anxiety, suicide, dizziness, pain, altered vision, encephalopathy and photophobia are summarised in Table 3. The results of the sensitivity analysis using the inverse variance method were in general similar to the results of the main meta-analysis in terms of the point estimate of prevalence, confidence interval boundaries and heterogeneity (Supplementary Table 3). An additional sensitivity analysis excluded studies that differed from the majority based on study design, retrospective or prospective data collection, atypical inclusion criteria, method of neuropsychiatric diagnosis and temporality of neuropsychiatric manifestations. There was no significant difference in pooled prevalence estimates based on four eligible symptoms (headache, encephalitis, myalgia and seizure) when excluding methodologically heterogeneous studies (Supplementary Figure 1).

**Table 4:** Pooled prevalence of individual neurological and psychiatric presentations.

Clinical feature	Pooled Prevalence (%)	CI (%)	Number of individuals (n)	Number of studies	Heterogeneity (%)
Seizure	2.7	0.7–10.2	74	2	0
Confusion	2.4	1.1–5.2	250	2	0
Encephalitis	2.0	0.5–8.2	283	3	55.8

### Secondary analysis

**Figure 3:**
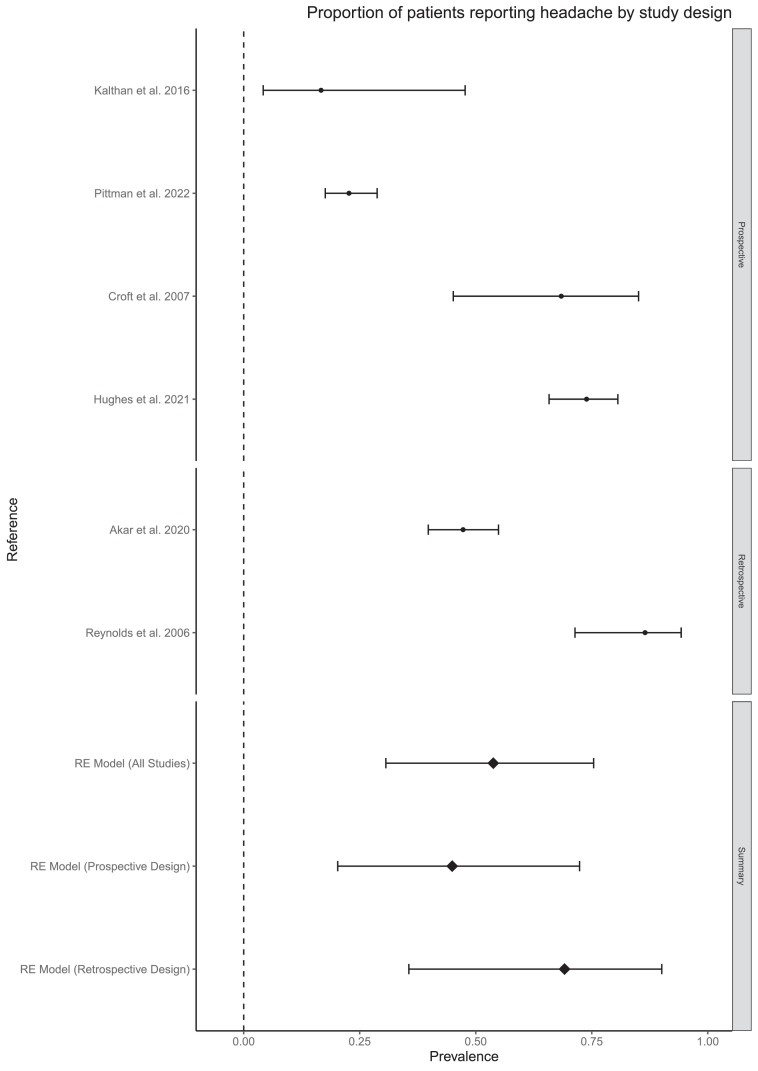
Subgroup analysis of headache.

There was no statistical evidence for a difference in the prevalence of headache in prospective compared to retrospective studies (based on four and two studies respectively, Figure 3). It was not possible to analyse subgroups based on illness severity or method of diagnosis due to missing data and lack of variation between groups. It was also not possible to pool high quality studies into a sensitivity analysis because there were no outcomes for which there existed more than one high quality study. No other clinical features met our prespecified eligibility criteria for subgroup analysis. However, other possible explanations for the heterogeneity across outcomes are clade of the virus, changes in symptom expression over time, illness severity, varying inclusion criteria, prospective vs retrospective symptom ascertainment and timing of symptom ascertainment relative to acute illness.

## Discussion

This systematic review and meta-analysis provides the first comprehensive overview of the prevalence of neurological and psychiatric presentations of MPX infection. Based on a small number of studies examining this topic, our meta-analysis found that serious neurological manifestations - seizure, confusion and encephalitis - were present in small (<3%) but non-negligible proportions of infected individuals. The prevalence of other neuropsychiatric symptoms including myalgia, fatigue, headache, anxiety and depression are less clear, but several studies found at least half of individuals experiencing some of these outcomes. There are also knowledge gaps surrounding putative factors that influence risk of neurological and psychiatric presentations including overall MPX illness severity and viral clade.

The prevalence of encephalitis in this population is likely higher than in the general population where the incidence of viral or unspecified encephalitis ranges from 0.5 to 16 cases/100,000/year.^36^ The relatively high prevalence of non-specific symptoms such as headache, myalgia and fatigue in some studies is perhaps unsurprising given that these symptoms are common in viral infections.^37^, ^38^, ^39^ It is likely that these symptoms represent a reaction to systemic illness rather than direct neurological injury. Additionally, a paucity of follow-up and lack of evaluation of symptom severity and timing makes it hard to ascertain whether these symptoms are potentially highly disabling or milder and/or transient. These findings are consistent with a recently published review of MPX epidemiology which found that fatigue/asthenia and headache were present in over a fifth of individuals and myalgia in slightly fewer.^40^ Although less frequently assessed, there was some evidence of psychiatric symptoms in people with MPX. For example, Ogoina and colleagues^13^ found that psychiatric manifestations including anxiety and depression were present in over a quarter of individuals hospitalised with MPX in Nigeria. Similarly, a case series from specialist centres in the UK found that three of seven patients admitted to hospital suffered from low mood.^5^ Although severity data was not reported, in both studies individuals with psychiatric symptoms required inpatient psychological therapies. It is important to note that anxiety and depression are common in hospitalised patients, and indeed in those who are in quarantine for infectious diseases, and the majority of individuals in this review were drawn from quarantined hospitalised samples.^41^^,^^42^ This evidence could point to an underrecognized and understudied burden of psychiatric complications in the acute phase of MPX.

The quality of evidence included in this review may reflect the relatively understudied phenomena of nervous system presentations in the context of MPX and affects what conclusions can be drawn. The majority of evidence was drawn from medium-low quality cohort and cross-sectional studies. Only one study included a control group, where individuals with Varicella zoster virus (VZV) and MPX were compared to those with MPX only and with VZV only.^25^ Individuals with MPX and VZV co-infection were more likely to report fatigue than those with VZV alone; however, no comparative data were given for the MPX only group and no other neuropsychiatric symptoms were compared. However, the clinical manifestations of VZV and MPX co-infection are complicated and the differences between individuals with co-infection and those with VZV alone may not be solely attributable to the effect of MPX. The effect of comorbid viral infection could also confound any potential association with MPX and neuropsychiatric presentations. For example, Ogoina and colleagues^13^ noted that nearly a quarter of included patients had comorbid HIV infection. Attributing causality of viral infection to neurological symptoms is difficult, despite established criteria used to define it.^43^ This is exacerbated by a lack of adequate comparison groups, such as healthy controls or individuals with other viral illnesses. The majority of studies which reported temporality of neuropsychiatric presentations stated that they occurred in the (sub)acute phase of MPX illness. However, a lack of reporting of premorbid neuropsychiatric diagnoses could conflate those that develop during or after MPX infection and prevents incidence from being ascertained. Additionally, the effect of comorbidity on the presence of neuropsychiatric clinical features is important to consider. However, due to a lack of sufficient reporting, we were unable to evaluate this.

The implications of our findings are somewhat restricted by the small sample size of the included studies, the actual number of included studies and the fact that prevalence estimates in the meta-analysis were derived from only 2 or 3 papers. For example, pooled prevalence of seizure was based on only 2 studies with a total of 74 patients. There was significant statistical and methodological heterogeneity between studies. In particular, half of the included studies were retrospective and relied on case note review, which risks a systematic under-representation of symptoms, especially, if neuropsychiatric features were not routinely inquired about or assessed, which is perhaps particularly pertinent with some of the milder symptoms we report. Furthermore, there is evidence that the evolution of MPX virus has accelerated recently^44^ and this may account for varying prevalences over time. Additionally, no studies included in this review assessed psychiatric symptoms using standardised scales and there was no description of clinical methods used to diagnose confusion or encephalopathy. The clinical significance of these symptoms is thus difficult to ascertain. In terms of data synthesis, we were limited by a lack of reporting of certain variables including MPX severity, ethnicity, and clade of MPX. The small number of studies means that subgroup analysis should be considered purely exploratory. Limited reporting of neurological investigations such as CSF analysis and neuroimaging also hinder understanding of the pathogenesis and potential mechanisms underlying the presentations described. For example, only one study reported neuroimaging findings from a patient with encephalitis.^26^

Although there has been little experimental work conducted on MPX and the nervous system in humans, a small number of case reports looking at smallpox have pointed to several diverse mechanistic explanations. Post-mortem examination revealed acute perivenular demyelination in patients known to have died of smallpox.^45^ Additionally, MRI scans in those with post-vaccination encephalitis have been suggestive of acute disseminated encephalomyelitis (ADEM).^10^ However, to date CSF from patients with post-vaccine CNS complications has overwhelmingly been normal with no viral load detected, consistent with aseptic meningitis,^9^ pointing to an immune-mediated pathogenesis. However, caution is required in extrapolating from either variola or vaccinia effects or neuropathology to MPX, despite shared genetics and clinical overlap between these *Orthopoxviruses* and their respective clinical syndromes. One case report included in this review of a child with MPX encephalitis, could not isolate viral material from CSF but did detect MPX specific IgM antibodies in CSF.^26^ This may suggest an intrathecal immune-mediated response; however, other cases of MPX-encephalitis did not report results of CSF analysis.^13^ Potential underlying mechanisms of MPX neuropsychiatric manifestations include a direct CNS infection, an immune-mediated response and a psychological reaction to illness.

Stigma could play a role in maladaptive psychological processes in those with MPX. Several studies emphasise the stigma associated with a diagnosis of MPX both on the individual and their family, affecting their integration back into society. Low mood was a common feature seen among many infected with MPX.^13^ One patient died from suicide a few days after admission. The reports cited concerns regarding how he had contracted MPX, and the effects on both him and his family.^13^ Others highlight the stigma associated with the focus on transmission related to close physical and sexual contact, which may place a potentially harmful emphasis on the LGBTQ+ community. Bragazzi and colleages^40^ point out the potential for exacerbation of stigma in already-stigmatised communities. Contemporary public health and education should make clear that although there have been a high number of cases in men who have sex with men, and some cases of MPX with co-infection of HIV/AIDS,^15^ MPX can also be spread via direct contact, clothing, and respiratory secretions, and that anyone can become infected.

Viral infections are known to have profound psychological effects on those affected, such as fear, loss, discrimination and stigma.^46^ Though the clinical course varies amongst individuals, a common progression of dermatological change is persistent scarring. Ogoina and colleagues^13^ report that not only were skin lesions widespread, itchy and tender causing disfigurement, but that some patients developed genital ulcers which were particularly distressing. A prior meta-analysis indicates a significant burden of persistent anxiety and depression in patients with facial scarring.^47^ In addition, Rumsey and Harcourt^48^ highlight the wider negative consequences such as reduced self-esteem and loss of identity. Whilst the studies included in this review focus on acute psychological symptoms, the long-term psychological consequences of MPX infection are unknown.

Similarly, it is unclear from the present study what the long-term outcomes for patients with MPX encephalitis are, aside from one reported death.^30^ Given that encephalitis, of infectious or autoimmune aetiology, results in considerable neurological and neuropsychiatric morbidity,^49^ collecting longitudinal data on affected individuals with this rare complication should be a high priority moving forward. The long-term neurocognitive effects of MPX infection also remain elusive. Pittman and colleagues^32^ reported a case of confusion and lethargy still present at discharge. Given the range of neuropsychiatric effects that occur in a proportion of people after several viral illnesses^50^, ^51^, ^52^ it may be worthwhile ascertaining whether these symptoms persist in MPX.

This paper has research and therapeutic implications. The variability in detection and reporting of neuropsychiatric manifestations highlights the need for registries of emerging zoonotic infections where clinicians can provide case histories and reliable data in rapidly evolving epidemics such as the WHO clinical data platform.^53^ The CoroNerve surveillance study^54^ demonstrates the utility of rapid reporting, having proved successful in the COVID-19 pandemic. Aside from epidemiology, there are therapeutic implications of this review. Our results suggest it would be worthwhile researching the value of integrating psychological support into the care of those isolated with MPX both in the acute setting and beyond, including those managed in the community. The inclusion of encephalitis as well as the psychosocial and emotional impacts for patients of contracting MPX will likely have implications for patient quality of life and therefore increased research in this field is an important area yet to be adequately addressed for patients and their caregivers/families.

There is preliminary evidence for a range of neurological and psychiatric presentations of MPX.

Seizure, encephalitis and confusion are present in a small proportion of infected individuals and several other clinical features may be common, though there is insufficient evidence to estimate their prevalence at present, as studies are sparse and highly heterogeneous. There is less evidence regarding the psychiatric sequelae of MPX, and although there are multiple reports of anxiety and depression, the prevalence of these symptoms is unknown. This preliminary suspicion that there are MPX-related nervous system presentations may warrant surveillance within the current MPX outbreak, with prospective longitudinal studies evaluating the mid- to long-term sequelae of the virus and well-powered prospective longitudinal studies to evaluate multi-system MPX effects. Robust methods to evaluate the potential causality of MPX infection with these manifestations are required at an individual and epidemiological level.

## Contributions

JB, TP and JPR conceptualised study design. JB, IC, CJW, ER and ZH conducted database searches and data extraction. Meta-analysis was conducted by CJW. The manuscript was written by JB, IC, ER, CJW, TP and JPR, and reviewed and edited by MB, AR, MSZ, GL, ASD, CFH, AE, BDM, TN and BC. JR and TP supervised and oversaw the project. All authors had full access to all the data included in this study and included data was verified by JB and IC. All authors had final responsibility for the decision to submit for publication.

## Data sharing statement

Data analysis code can be found in the supplementary methods and at the following link: https://github.com/CameronWatson2020/monkeypox/blob/main/monkeypox_analysis.R.

## Declaration of interests

All authors have completed ICMJE uniform disclosure forms and declare; GL is supported by the UCLH BRC, is funded by NIHR, and is TSC chair for NIHR study and Wellcome Clinical PhD funding for JPR. CW receives support from the Royal College of Psychiatrists Pathfinder Fellowship and the Association of British Neurologists' Bursary. AE is a recipient of various grants for The Encephalitis Society which she is chief executive of, she has received payment for speaking and presentations from Pfizer, UCB, Bavarian Nordics, Valneva, CSL Behring and Biomerieux. MZ was supported to attend the European Academy of Neurology 2022 Encephalitis Workshop and Eisai Dec 2019 one lecture honoraria. ZH was supported to attend meetings by the Royal College of Psychiatrists Foundation Fellowship. BDM is supported by the UKRI/MRC (MR/V03605X/1), the MRCCSF (MR/V007181/1), the MRC/AMED (MR/T028750/1) and the Wellcome Trust (102186/B/13/Z).

No other relationships or activities that could appear to have influenced the submitted work.

## Appendix Supplementary materials

Image, application 1

Image, application 2

Image, application 3

Image, application 4

Image, application 5

Image, application 6

Image, application 7

Image, application 8

Image, application 9
