# Supplementary figures and images for: Neurological and psychiatric presentations associated with human monkeypox virus infection: A systematic review and meta-analysis
Source: eClinicalMedicine. 2022 Sep 8;52:101644. doi: 10.1016/j.eclinm.2022.101644 (PMC9533950; doi:10.1016/j.eclinm.2022.101644)

Author(s) and Year

Encephalitis N

Proportion [95% CI]

Huhn et al. 2005

1 34

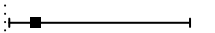

0.03 [0.00, 0.18]

Ogoina et al. 2020

3 40

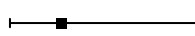

0.07 [0.02, 0.21]

( $I^2 = 0.0\%$ )

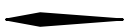

0.05 [0.02, 0.14]

0 0.05 0.15 0.25

Proportion

Supplement: Supplementary file 1 [file mmc1.pdf]

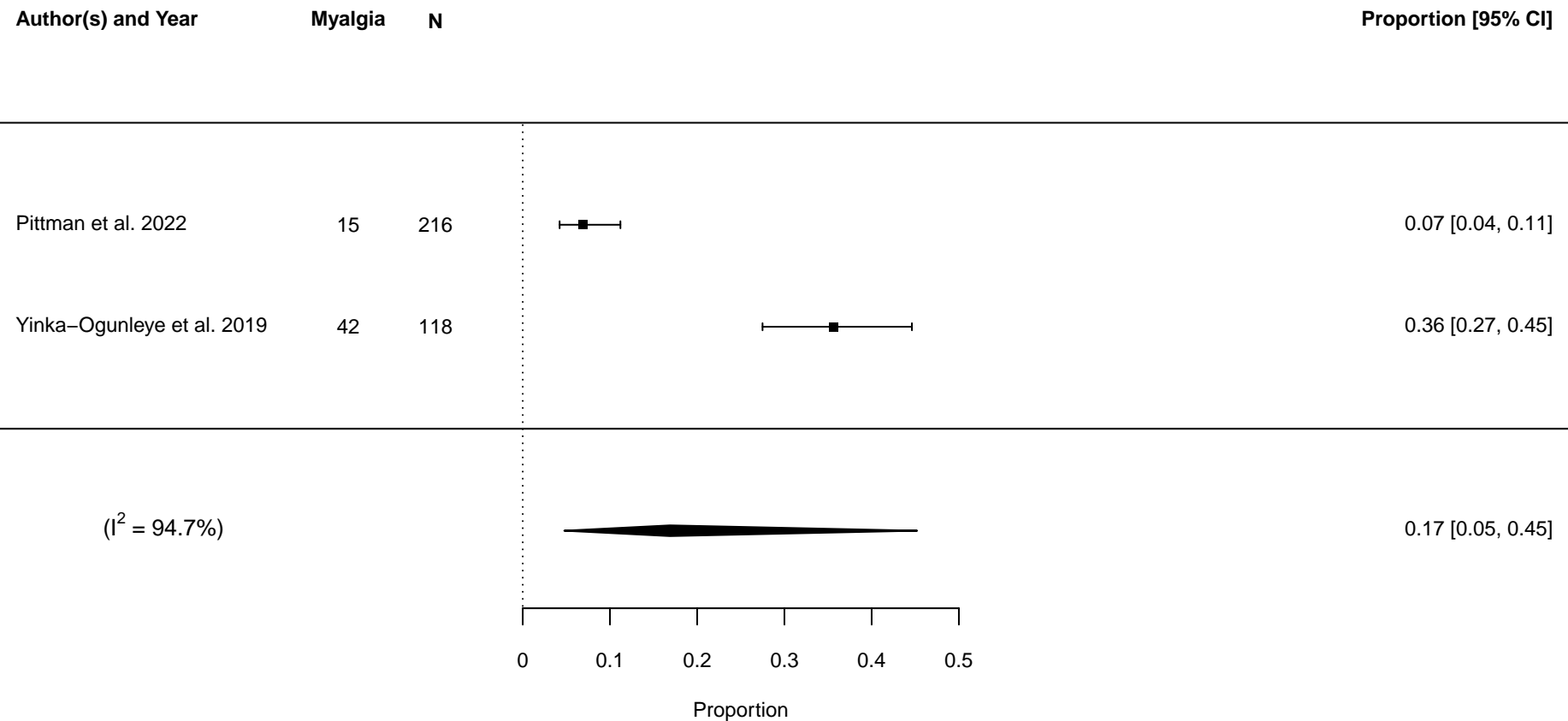

Supplement: Supplementary file 3 [file mmc3.pdf]

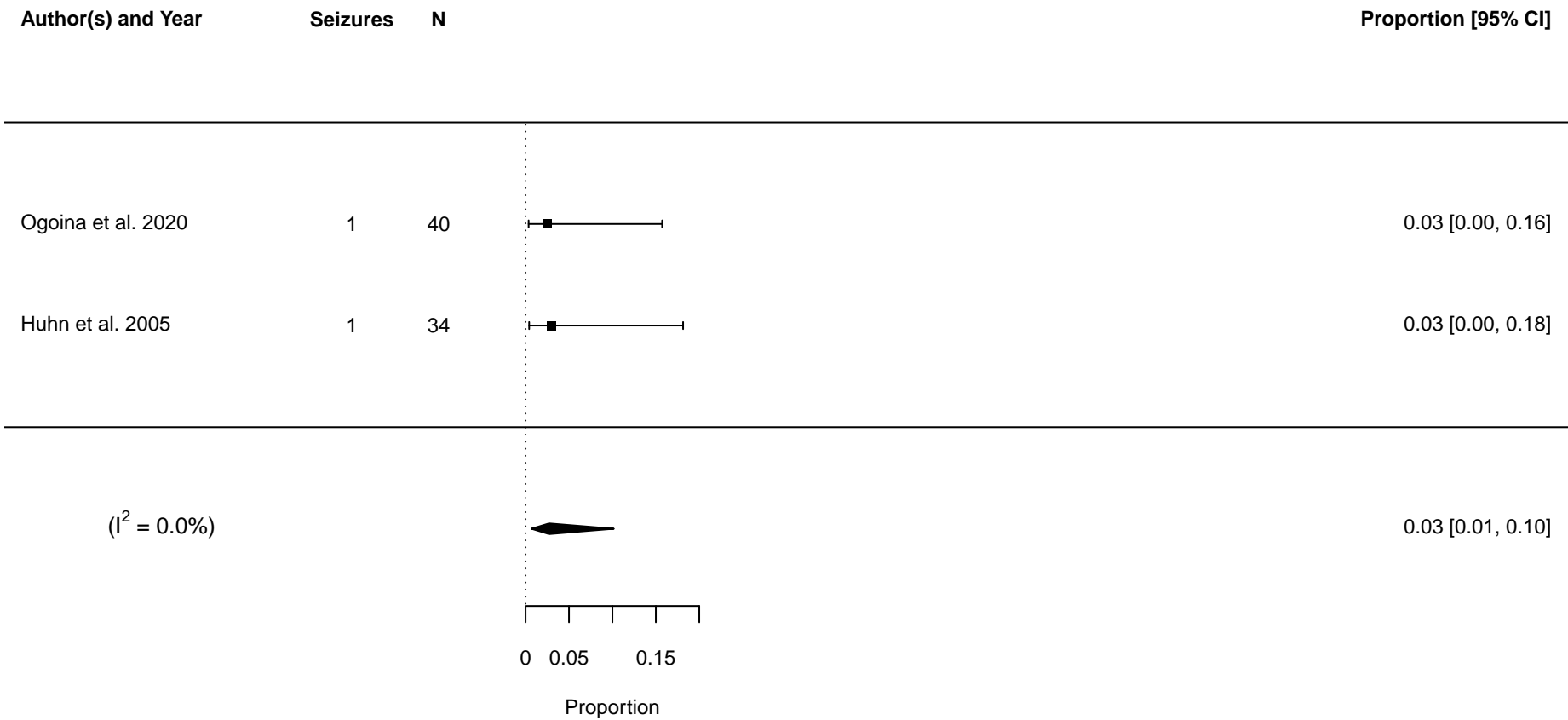

Supplement: Supplementary file 4 [file mmc4.pdf]
