# Supplementary material for: Neurological and psychiatric presentations associated with human monkeypox virus infection: A systematic review and meta-analysis
Source: eClinicalMedicine. 2022 Sep 8;52:101644. doi: 10.1016/j.eclinm.2022.101644 (PMC9533950; doi:10.1016/j.eclinm.2022.101644)
Supplement: Supplementary file 2 [file mmc2.pdf]

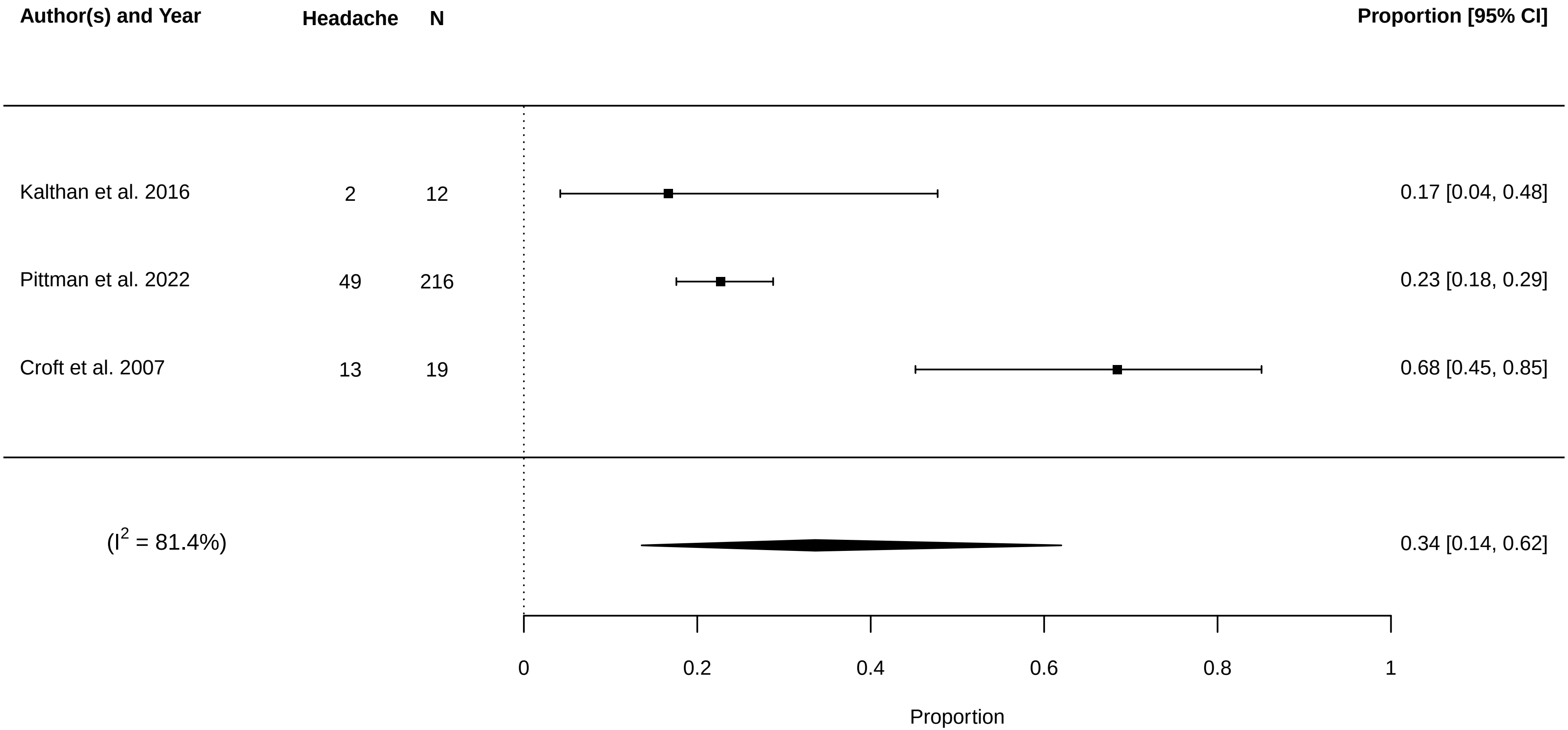
Author(s) and Year
Headache
N
Proportion [95% CI]
Kalthan et al. 2016
2
12
0.17 [0.04, 0.48]
Pittman et al. 2022
49
216
0.23 [0.18, 0.29]
Croft et al. 2007
13
19
0.68 [0.45, 0.85]
(I2 = 81.4%)
0.34 [0.14, 0.62]
0
0.2
0.4
0.6
0.8
1
Proportion
