# Supplementary material for: Neurological and psychiatric presentations associated with human monkeypox virus infection: A systematic review and meta-analysis
Source: eClinicalMedicine. 2022 Sep 8;52:101644. doi: 10.1016/j.eclinm.2022.101644 (PMC9533950; doi:10.1016/j.eclinm.2022.101644)
Supplement: Supplementary file 5 [file mmc5.docx]

**Supplementary Methods Data analysis code**

#------ Generalised Linear Mixed Models for monkeypox meta-analyses ------ #

# code produced by C.Watson based on analysis in Badenoch et al., 2022.

# last updated on 15/07/22 by C.Watson (sensitivity analysis comments added)

# this code contains all meta-analytic calculations for the paper:

# "Neurological and psychiatric presentations associated with human monkeypox virus infection: a systematic review and meta-analysis"

# The code is divided into four major sections:

# PREPARATION: Here, the packages necessary for the analysis are loaded

# and the data frame is prepared for the analysis.

# MAIN ANALYSIS: In this section, the main analyses of the different symptoms are conducted

# using generalized linear mixed models. Forest plots for all symptoms are produced

# SUBGROUP ANALYSIS: This section contains the subgroup analysis of the symptom "headache" depending on

# whether the study was retrospective or prospective in design

# z-values and p-values are computed for the subgroup comparison.

# finally, a forest plot of the subgroup analysis is produced.

# SENSITIVITY ANALYSIS: In this section, the main analyses are repeated using an inverse-variance model

# with the Freeman-Tukey double-arcsine transformation. This is done as a sensitivity analysis.

# We then conduct a meta-analysis using on homogenous studies

## PREPARATION -------------------------------------------------------------------------------------------------------------------

# Load packages ------------------------------------------------------------------------------------------------------------------

library(dplyr)

library(metafor)

library(readr)

library(readxl)

library(ggplot2)

library(tidyverse)

# set working directory ----------------------------------------------------------------------------------------------------------

# set working directory manually

setwd("~/Documents/Medicine/Research/Monkeypox//")

csv_name <- "Monkeypox data extraction - sheet for analysis.csv"

meta_data <- read_csv(csv_name)

meta_data$n <- as.numeric(as.character(meta_data$n)

## MAIN ANALYSIS -----------------------------------------------------------------------------------------------

# 1: Headache - Full meta-analysis - 6 studies ------------------------------------------

# prepare data frame for meta-analysis

meta_data$headache <- as.numeric(as.character(meta_data$headache))

head_df <- meta_data %>% filter(!is.na(headache))

head_df <- head_df %>% mutate(head_prop = headache/n)

head_df <- head_df %>% arrange(head_prop)

# estimate glmm for full meta-analysis

head_glmm <- rma.glmm(xi = headache, ni = n, data = head_df,

slab = paste(reference),

measure = "PLO")

head_glmm

# get prevalence estimates

predict(head_glmm, transf = transf.ilogit)

# get sample size

sum(head_df$n)

# draw forest plot for headache meta-analysis

forest(head_glmm,

xlim=c(-0.6,1.2),

ilab=cbind(head_df$headache, head_df$n), ilab.xpos=c(-0.2,-0.1),

transf = transf.ilogit,

cex=.75, header="Author(s) and Year", mlab="",)

op <- par(cex=.75, font=2)

text(c(-0.2,-0.1), 8.04, c("Headache", "N"))

par(op)

text(-0.5, -1, pos=4, cex=0.8, bquote(paste("(", I^2, " = ",

.(formatC(head_glmm$I2, digits=1, format="f")), "%)")))

#1a - Headache - Sub-group Analysis

all_head <- rma.glmm(xi = headache, ni = n, data = head_df,

slab = paste(reference),

measure = "PLO")

prospo_head <- rma.glmm(xi = headache, ni = n, data = head_df,

subset = (design == "Prospective"),

slab = paste(reference),

measure = "PLO")

retro_head <- rma.glmm(xi = headache, ni = n, data = head_df,

subset = (design == "Retrospective"),

slab = paste(reference),

measure = "PLO")

# create plot for subgroup analysis --------------------------

plot_data <- data.frame(reference = attr(all_head$yi, "slab"), yi = all_head$yi, se = sqrt(all_head$vi))

plot_data$ci.lb = plot_data$yi - qnorm(1 - 0.05/2)*plot_data$se

plot_data$ci.ub = plot_data$yi + qnorm(1 - 0.05/2)*plot_data$se

data <- head_df %>% mutate(reference = paste(reference))

design_data <- data %>%

select(reference, design)

plot_data <- inner_join(plot_data, design_data, by = "reference")

n_rows <- nrow(plot_data)

plot_data$I2 = rep(NA, n_rows)

plot_data$summary = rep("No", n_rows)

plot_data$plot_order <- order(-plot_data$yi)

names(plot_data)

summary_data <- data.frame(reference = c("RE Model (All Studies)","RE Model (Prospective Design)",

"RE Model (Retrospective Design)"),

yi = c(all_head$b, prospo_head$b, retro_head$b),

se = c(all_head$se, prospo_head$se, retro_head$se),

ci.lb = c(all_head$ci.lb, prospo_head$ci.lb, retro_head$ci.lb),

ci.ub = c(all_head$ci.ub, prospo_head$ci.ub, retro_head$ci.ub),

design = c("Summary", "Summary", "Summary"),

I2 = c(all_head$I2, prospo_head$I2, retro_head$I2),

summary = rep("Yes", 3),

plot_order = -1:-3

)

plot_data <- bind_rows(plot_data, summary_data)

plot_data$yi_prev = transf.ilogit(plot_data$yi)

plot_data$ci.lb_prev = transf.ilogit(plot_data$ci.lb)

plot_data$ci.ub_prev = transf.ilogit(plot_data$ci.ub)

apatheme=theme_bw()+

theme(panel.grid.major=element_blank(),

panel.grid.minor=element_blank(),

panel.border=element_blank(),

axis.line=element_line(),

legend.position='none')

plot = ggplot(plot_data, aes(y = reorder(reference, plot_order), x = yi_prev, xmin = ci.lb_prev, xmax = ci.ub_prev, shape = summary))+

geom_point(color = "black")+

geom_point(data = subset(plot_data, summary=="Yes"), color ='black', shape=18, size = 4)+

geom_errorbarh(height = .1)+

scale_x_continuous(limits = c(-0.05, 1.0), name = "Prevalence")+

ylab("Reference")+

geom_vline(xintercept = 0, color = "black", linetype = "dashed")+

facet_grid(design ~., scales = "free", space = "free")+

ggtitle("Proportion of patients reporting headache by study design") +

apatheme+

theme(strip.text.y = element_text(size = 7),

plot.title = element_text(hjust = 1))

plot

all_head

prospo_head

predict(prospo_head, transf = transf.ilogit)

retro_head

predict(retro_head, transf = transf.ilogit)

# calculate z and p values for the comparison

zval_head = (coef(retro_head)-coef(prospo_head))/sqrt(retro_head$se^2+prospo_head$se^2)

pval_head = 2*pnorm(abs(zval_head), lower.tail = FALSE)

zval_head

pval_head

# 2: Myalgia - Full meta-analysis - 4 studies ------------------------------------------

# prepare data frame for meta-analysis

meta_data$myalgia <- as.numeric(as.character(meta_data$myalgia))

myalgia_df <- meta_data %>% filter(!is.na(myalgia))

myalgia_df <- myalgia_df %>% mutate(myalgia_prop = myalgia/n)

myalgia_df <- myalgia_df %>% arrange(myalgia_prop)

# estimate glmm for full meta-analysis

myalgia_glmm <- rma.glmm(xi = myalgia, ni = n, data = myalgia_df,

slab = paste(reference),

measure = "PLO")

myalgia_glmm

# get prevalence estimates

predict(myalgia_glmm, transf = transf.ilogit)

# get sample size

sum(myalgia_df$n)

# draw forest plot for myalgia meta-analysis

pdf(file="myalgia_full.pdf", width = 11)

forest(myalgia_glmm,

xlim=c(-0.6,1.2),

ilab=cbind(myalgia_df$myalgia, myalgia_df$n), ilab.xpos=c(-0.2,-0.1),

transf = transf.ilogit,

cex=.75, header="Author(s) and Year", mlab="",)

op <- par(cex=.75, font=2)

text(c(-0.2,-0.1), 6, c("Myalgia", "N"))

par(op)

text(-0.5, -1, pos=4, cex=0.8, bquote(paste("(", I^2, " = ",

.(formatC(myalgia_glmm$I2, digits=1, format="f")), "%)")))

dev.off()

# 3: Seizures - Full meta-analysis - 2 studies ------------------------------------------

# prepare data frame for meta-analysis

meta_data$seizure <- as.numeric(as.character(meta_data$seizure))

seizure_df <- meta_data %>% filter(!is.na(seizure))

seizure_df <- seizure_df %>% mutate(seizure_prop = seizure/n)

seizure_df <- seizure_df %>% arrange(seizure_prop)

# estimate glmm for full meta-analysis

seizure_glmm <- rma.glmm(xi = seizure, ni = n, data = seizure_df,

slab = paste(reference),

measure = "PLO")

seizure_glmm

# get prevalence estimates

predict(seizure_glmm, transf = transf.ilogit)

# get sample size

sum(seizure_df$n)

# draw forest plot for seizure meta-analysis

forest(seizure_glmm,

xlim=c(-0.6,1.2),

ilab=cbind(seizure_df$seizure, seizure_df$n), ilab.xpos=c(-0.2,-0.1),

transf = transf.ilogit,

cex=.75, header="Author(s) and Year", mlab="",)

op <- par(cex=.75, font=2)

text(c(-0.2,-0.1), 4.02, c("Seizures", "N"))

par(op)

text(-0.5, -1, pos=4, cex=0.8, bquote(paste("(", I^2, " = ",

.(formatC(seizure_glmm$I2, digits=1, format="f")), "%)")))

# 4: Confusion - Full meta-analysis - 2 studies ------------------------------------------

# prepare data frame for meta-analysis

meta_data$confusion <- as.numeric(as.character(meta_data$confusion))

confusion_df <- meta_data %>% filter(!is.na(confusion))

confusion_df <- confusion_df %>% mutate(confusion_prop = confusion/n)

confusion_df <- confusion_df %>% arrange(confusion_prop)

# estimate glmm for full meta-analysis

confusion_glmm <- rma.glmm(xi = confusion, ni = n, data = confusion_df,

slab = paste(reference),

measure = "PLO")

confusion_glmm

# get prevalence estimates

predict(confusion_glmm, transf = transf.ilogit)

# get sample size

sum(confusion_df$n)

# draw forest plot for confusion meta-analysis

forest(confusion_glmm,

xlim=c(-0.6,1.2),

ilab=cbind(confusion_df$confusion, confusion_df$n), ilab.xpos=c(-0.2,-0.1),

transf = transf.ilogit,

cex=.75, header="Author(s) and Year", mlab="",)

op <- par(cex=.75, font=2)

text(c(-0.2,-0.1), 4.02, c("Confusion", "N"))

par(op)

text(-0.5, -1, pos=4, cex=0.8, bquote(paste("(", I^2, " = ",

.(formatC(confusion_glmm$I2, digits=1, format="f")), "%)")))

# 5: Encephalitis - Full meta-analysis - 3 studies ------------------------------------------

# prepare data frame for meta-analysis

meta_data$encephalitis <- as.numeric(as.character(meta_data$encephalitis))

enceph_df <- meta_data %>% filter(!is.na(encephalitis))

enceph_df <- enceph_df %>% mutate(enceph_prop = encephalitis/n)

enceph_df <- enceph_df %>% arrange(enceph_prop)

# estimate glmm for full meta-analysis

enceph_glmm <- rma.glmm(xi = encephalitis, ni = n, data = enceph_df,

slab = paste(reference),

measure = "PLO")

enceph_glmm

# get prevalence estimates

predict(enceph_glmm, transf = transf.ilogit)

# get sample size

sum(enceph_df$n)

# draw forest plot for encephalitis meta-analysis

forest(enceph_glmm,

xlim=c(-0.6,1.2),

ilab=cbind(enceph_df$encephalitis, enceph_df$n), ilab.xpos=c(-0.2,-0.1),

transf = transf.ilogit,

cex=.75, header="Author(s) and Year", mlab="",)

op <- par(cex=.75, font=2)

text(c(-0.2,-0.1), 5, c("Encephalitis", "N"))

par(op)

text(-0.5, -1, pos=4, cex=0.8, bquote(paste("(", I^2, " = ",

.(formatC(enceph_glmm$I2, digits=1, format="f")), "%)")))

# 6: Fatigue - Full meta-analysis - 2 studies ------------------------------------------

# prepare data frame for meta-analysis

meta_data$fatigue <- as.numeric(as.character(meta_data$fatigue))

fatigue_df <- meta_data %>% filter(!is.na(fatigue))

fatigue_df <- fatigue_df %>% mutate(fatigue_prop = fatigue/n)

fatigue_df <- fatigue_df %>% arrange(fatigue_prop)

# estimate glmm for full meta-analysis

fatigue_glmm <- rma.glmm(xi = fatigue, ni = n, data = fatigue_df,

slab = paste(reference),

measure = "PLO")

fatigue_glmm

# get prevalence estimates

predict(fatigue_glmm, transf = transf.ilogit)

# get sample size

sum(fatigue_df$n)

# draw forest plot for fatigue meta-analysis

forest(fatigue_glmm,

xlim=c(-0.6,1.2),

ilab=cbind(fatigue_df$fatigue, fatigue_df$n), ilab.xpos=c(-0.2,-0.1),

transf = transf.ilogit,

cex=.75, header="Author(s) and Year", mlab="",)

op <- par(cex=.75, font=2)

text(c(-0.2,-0.1), 4.02, c("Fatigue", "N"))

par(op)

text(-0.5, -1, pos=4, cex=0.8, bquote(paste("(", I^2, " = ",

.(formatC(fatigue_glmm$I2, digits=1, format="f")), "%)")))

## SENSITIVITY ANALYSIS ----------------------------------------------------------------------------------------

# 1 - Headache

sensitivity_head <- rma.uni(xi = headache, ni = n, data = head_df,

slab = paste(reference),

measure = "PFT",

method = "REML")

sensitivity_head

predict(sensitivity_head, transf=transf.ipft.hm, targs=list(ni=sensitivity_head$ni))

# 2 - Myalgia

sensitivity_myalgia <- rma.uni(xi = myalgia, ni = n, data = myalgia_df,

slab = paste(reference),

measure = "PFT",

method = "REML")

sensitivity_myalgia

predict(sensitivity_myalgia, transf=transf.ipft.hm, targs=list(ni=sensitivity_myalgia$ni))

# 3 - Seizures

sensitivity_seizures <- rma.uni(xi = seizure, ni = n, data = seizure_df,

slab = paste(reference),

measure = "PFT",

method = "REML")

sensitivity_seizures

predict(sensitivity_seizures, transf=transf.ipft.hm, targs=list(ni=sensitivity_seizures$ni))

# 4 - Confusion

sensitivity_confusion <- rma.uni(xi = confusion, ni = n, data = confusion_df,

slab = paste(reference),

measure = "PFT",

method = "REML")

sensitivity_confusion

predict(sensitivity_confusion, transf=transf.ipft.hm, targs=list(ni=sensitivity_confusion$ni))

# 5 - Encephalitis

sensitivity_enceph <- rma.uni(xi = encephalitis, ni = n, data = enceph_df,

slab = paste(reference),

measure = "PFT",

method = "REML")

sensitivity_enceph

predict(sensitivity_enceph, transf=transf.ipft.hm, targs=list(ni=sensitivity_enceph$ni))

# 6 - Fatigue

sensitivity_fat <- rma.uni(xi = fatigue, ni = n, data = fatigue_df,

slab = paste(reference),

measure = "PFT",

method = "REML")

sensitivity_fat

predict(sensitivity_fat, transf=transf.ipft.hm, targs=list(ni=sensitivity_fat$ni))

# Homogeneity analysis

csv_name <- "Monkeypox data extraction - Homogeniety analysis.csv"

meta_data <- read_csv(csv_name)

meta_data$n <- as.numeric(as.character(meta_data$n)

# Headache

# prepare data frame for meta-analysis

meta_data$headache <- as.numeric(as.character(meta_data$headache))

head_df <- meta_data %>% filter(!is.na(headache))

head_df <- head_df %>% mutate(head_prop = headache/n)

head_df <- head_df %>% arrange(head_prop)

# estimate glmm for full meta-analysis

head_glmm <- rma.glmm(xi = headache, ni = n, data = head_df,

slab = paste(reference),

measure = "PLO")

head_glmm

# get prevalence estimates

predict(head_glmm, transf = transf.ilogit)

# get sample size

sum(head_df$n)

forest(head_glmm,

xlim=c(-0.6,1.2),

ilab=cbind(head_df$headache, head_df$n), ilab.xpos=c(-0.2,-0.1),

transf = transf.ilogit,

cex=.75, header="Author(s) and Year", mlab="",)

op <- par(cex=.75, font=2)

text(c(-0.2,-0.1), 5, c("Headache", "N"))

par(op)

text(-0.5, -1, pos=4, cex=0.8, bquote(paste("(", I^2, " = ",

.(formatC(head_glmm$I2, digits=1, format="f")), "%)")))

# Myalgia

# prepare data frame for meta-analysis

meta_data$myalgia <- as.numeric(as.character(meta_data$myalgia))

myalgia_df <- meta_data %>% filter(!is.na(myalgia))

myalgia_df <- myalgia_df %>% mutate(myalgia_prop = myalgia/n)

myalgia_df <- myalgia_df %>% arrange(myalgia_prop)

# estimate glmm for full meta-analysis

myalgia_glmm <- rma.glmm(xi = myalgia, ni = n, data = myalgia_df,

slab = paste(reference),

measure = "PLO")

myalgia_glmm

# get prevalence estimates

predict(myalgia_glmm, transf = transf.ilogit)

# get sample size

sum(myalgia_df$n)

forest(myalgia_glmm,

xlim=c(-0.6,1.2),

ilab=cbind(myalgia_df$myalgia, myalgia_df$n), ilab.xpos=c(-0.2,-0.1),

transf = transf.ilogit,

cex=.75, header="Author(s) and Year", mlab="",)

op <- par(cex=.75, font=2)

text(c(-0.2,-0.1), 4, c("Myalgia", "N"))

par(op)

text(-0.5, -1, pos=4, cex=0.8, bquote(paste("(", I^2, " = ",

.(formatC(myalgia_glmm$I2, digits=1, format="f")), "%)")))

# Seizure

meta_data$seizure <- as.numeric(as.character(meta_data$seizure))

seizure_df <- meta_data %>% filter(!is.na(seizure))

seizure_df <- seizure_df %>% mutate(seizure_prop = seizure/n)

seizure_df <- seizure_df %>% arrange(seizure_prop)

# estimate glmm for full meta-analysis

seizure_glmm <- rma.glmm(xi = seizure, ni = n, data = seizure_df,

slab = paste(reference),

measure = "PLO")

seizure_glmm

# get prevalence estimates

predict(seizure_glmm, transf = transf.ilogit)

# get sample size

sum(seizure_df$n)

forest(seizure_glmm,

xlim=c(-0.6,1.2),

ilab=cbind(seizure_df$seizure, seizure_df$n), ilab.xpos=c(-0.2,-0.1),

transf = transf.ilogit,

cex=.75, header="Author(s) and Year", mlab="",)

op <- par(cex=.75, font=2)

text(c(-0.2,-0.1), 4.02, c("Seizures", "N"))

par(op)

text(-0.5, -1, pos=4, cex=0.8, bquote(paste("(", I^2, " = ",

.(formatC(seizure_glmm$I2, digits=1, format="f")), "%)")))

# Encephalitis

# prepare data frame for meta-analysis

meta_data$encephalitis <- as.numeric(as.character(meta_data$encephalitis))

enceph_df <- meta_data %>% filter(!is.na(encephalitis))

enceph_df <- enceph_df %>% mutate(enceph_prop = encephalitis/n)

enceph_df <- enceph_df %>% arrange(enceph_prop)

# estimate glmm for full meta-analysis

enceph_glmm <- rma.glmm(xi = encephalitis, ni = n, data = enceph_df,

slab = paste(reference),

measure = "PLO")

enceph_glmm

# get prevalence estimates

predict(enceph_glmm, transf = transf.ilogit)

# get sample size

sum(enceph_df$n)

forest(enceph_glmm,

xlim=c(-0.6,1.2),

ilab=cbind(enceph_df$encephalitis, enceph_df$n), ilab.xpos=c(-0.2,-0.1),

transf = transf.ilogit,

cex=.75, header="Author(s) and Year", mlab="",)

op <- par(cex=.75, font=2)

text(c(-0.2,-0.1), 4, c("Encephalitis", "N"))

par(op)

text(-0.5, -1, pos=4, cex=0.8, bquote(paste("(", I^2, " = ",

.(formatC(enceph_glmm$I2, digits=1, format="f")), "%)")))
