# Supplementary material for: Neurological and psychiatric presentations associated with human monkeypox virus infection: A systematic review and meta-analysis
Source: eClinicalMedicine. 2022 Sep 8;52:101644. doi: 10.1016/j.eclinm.2022.101644 (PMC9533950; doi:10.1016/j.eclinm.2022.101644)
Supplement: Supplementary file 6 [file mmc6.docx]

**Supplementary methods: full search strategy**

**Medline**

(Monkeypox OR monkeypox virus OR monkey pox OR MPV)

AND

((neurol* OR nervous OR brain OR CNS OR encephal* OR mening* OR myeli* OR myalg* OR ADEM OR ataxi* OR dysphasi* OR aphasi* OR stroke OR guillain-barre OR Miller-Fisher OR paresis OR palsy OR cerebr* OR crani* OR epilep* OR seizure or headache* OR migraine* OR demyeli* OR neuroimag* OR neurotrop* OR neuroinvas* OR neuropath* OR cerebrospinal* or cerebro-spinal OR CSF OR*deliri* OR sleep OR insomnia OR somnolence OR hypersomnolence OR parasomnia OR "movement disorder" OR neuropsych* OR dement* OR cogniti* OR irritability OR hallucinat* OR delusion* OR apath* OR indifference OR agitat* OR euphori* OR elation OR elated OR disinhibit* OR aggressi* OR amnes* OR catatoni* OR personality OR psycho* OR mental OR mood OR affective OR depress* OR anxi* OR "obsessive compulsive" OR OCD OR "panic disorder" OR post-trauma* OR posttrauma* OR PTSD OR neurosis OR neurotic OR bipolar OR mania OR manic OR schizophreni* OR "intelligence quotient" OR IQ OR "mental retardation" OR "intellectual disability" OR "learning disability" OR autis* OR asperger* OR "attention deficit" OR ADHD OR hyperactivity OR hyperkinetic OR suicid* OR emotion* OR appetite OR fatigu* OR tired* OR confus*).ti,ab

OR

(exp Neurology/ or exp Nervous System/ or exp Nervous System Diseases/ or exp Neurologic Manifestations/ or exp Psychiatry/ or exp Mental Processes/ or exp Behavioral Symptoms/ or exp Psychological Phenomena/ or exp DELIRIUM/ OR exp SLEEP/ OR exp WAKEFULNESS/ OR exp SLEEP/ OR exp "DISORDERS OF EXCESSIVE SOMNOLENCE"/ OR exp PARASOMNIAS/ OR exp "PSYCHOMOTOR DISORDERS"/ OR exp DEMENTIA/ OR exp "NEUROCOGNITIVE DISORDERS"/ OR exp HALLUCINATIONS/ OR exp DELUSIONS/ OR exp APATHY/ OR exp "PSYCHOMOTOR AGITATION"/ OR exp EUPHORIA/ OR exp AGGRESSION/ OR exp AMNESIA/ OR exp CATATONIA/ OR exp "PERSONALITY DISORDERS"/ OR exp "SCHIZOPHRENIA SPECTRUM AND OTHER PSYCHOTIC DISORDERS"/ OR exp "MENTAL DISORDERS"/ OR exp "MOOD DISORDERS"/ OR exp DEPRESSION/ OR exp ANXIETY/ OR exp "ANXIETY DISORDERS"/ OR exp "OBSESSIVECOMPULSIVE DISORDER"/ OR exp "PANIC DISORDER"/ OR exp "STRESS DISORDERS, POST-TRAUMATIC"/ OR exp "BIPOLAR AND RELATED DISORDERS"/ OR exp SCHIZOPHRENIA/ OR exp "INTELLECTUAL DISABILITY"/ OR exp "AUTISM SPECTRUM DISORDER"/ OR exp "ASPERGER SYNDROME"/ OR exp "ATTENTION DEFICIT AND DISRUPTIVE BEHAVIOR DISORDERS"/ OR exp "ATTENTION DEFICIT DISORDER WITH HYPERACTIVITY"/ OR exp "MOTOR ACTIVITY"/ OR exp SUICIDE/ OR exp EMOTIONS/ OR exp APPETITE/ OR exp "FEEDING AND EATING DISORDERS"/ OR exp FATIGUE/ OR exp CONFUSION/)) [Humans]

**Embase**

(Monkeypox OR monkeypox virus OR monkey pox OR MPV)

AND

((neurol* OR nervous OR brain OR CNS OR encephal* OR mening* OR myeli* OR myalg* OR ADEM OR ataxi* OR dysphasi* OR aphasi* OR stroke OR guillain-barre OR Miller-Fisher OR paresis OR palsy OR cerebr* OR crani* OR epilep* OR seizure or headache* OR migraine* OR demyeli* OR neuroimag* OR neurotrop* OR neuroinvas* OR neuropath* OR cerebrospinal* or cerebro-spinal OR CSF OR*deliri* OR sleep OR insomnia OR somnolence OR hypersomnolence OR parasomnia OR "movement disorder" OR neuropsych* OR dement* OR cogniti* OR irritability OR hallucinat* OR delusion* OR apath* OR indifference OR agitat* OR euphori* OR elation OR elated OR disinhibit* OR aggressi* OR amnes* OR catatoni* OR personality OR psycho* OR mental OR mood OR affective OR depress* OR anxi* OR "obsessive compulsive" OR OCD OR "panic disorder" OR post-trauma* OR posttrauma* OR PTSD OR neurosis OR neurotic OR bipolar OR mania OR manic OR schizophreni* OR "intelligence quotient" OR IQ OR "mental retardation" OR "intellectual disability" OR "learning disability" OR autis* OR asperger* OR "attention deficit" OR ADHD OR hyperactivity OR hyperkinetic OR suicid* OR emotion* OR appetite OR fatigu* OR tired* OR confus*).ti,ab OR

(exp Neuroscience/ or exp Nervous System/ or exp Neurologic Disease/ or exp Psychiatry/ or exp Behavior/ or exp Mental Function/ or exp Psychophysiology/ or exp DELIRIUM/ OR exp "SLEEP DISORDER"/ OR exp INSOMNIA/ OR exp SOMNOLENCE/ OR exp HYPERSOMNIA/ OR exp PARASOMNIA/ OR exp "MOTOR DYSFUNCTION"/ OR exp DEMENTIA/ OR exp "COGNITIVE DEFECT"/ OR exp IRRITABILITY/ OR exp HALLUCINATION/ OR exp DELUSION/ OR exp APATHY/ OR exp AGITATION/ OR exp EUPHORIA/ OR exp AGGRESSION/ OR exp AMNESIA/ OR exp CATATONIA/ OR exp "PERSONALITY DISORDER"/ OR exp PSYCHOSIS/ OR exp "MENTAL DISEASE"/ OR exp MOOD/ OR exp "MOOD DISORDER"/ OR exp DEPRESSION/ OR exp 7 ANXIETY/ OR exp "ANXIETY DISORDER"/ OR exp "OBSESSIVE COMPULSIVE DISORDER"/ OR exp PANIC/ OR exp "POSTTRAUMATIC STRESS DISORDER"/ OR exp NEUROSIS/ OR exp "BIPOLAR DISORDER"/ OR exp MANIA/ OR exp PSYCHOSIS/ OR exp "SCHIZOPHRENIA SPECTRUM DISORDER"/ OR exp SCHIZOPHRENIA/ OR exp "INTELLIGENCE QUOTIENT"/ OR exp "MENTAL DISEASE"/ OR exp "INTELLECTUAL IMPAIRMENT"/ OR exp "DISORDERS OF HIGHER CEREBRAL FUNCTION"/ OR exp "LEARNING DISORDER"/ OR exp AUTISM/ OR exp "ATTENTION DEFICIT DISORDER"/ OR exp HYPERACTIVITY/ OR exp "PSYCHOMOTOR DISORDER"/ OR exp HYPERKINESIA/ OR exp SUICIDE/ OR exp "SUICIDAL BEHAVIOR"/ OR exp "SUICIDE ATTEMPT"/ OR exp EMOTION/ OR exp APPETITE/ OR exp "APPETITE DISORDER"/ OR exp FATIGUE/ OR exp CONFUSION)) [Humans]

**Psychinfo**

(Monkeypox OR monkeypox virus OR monkey pox OR MPV)

AND

((neurol* OR nervous OR brain OR CNS OR encephal* OR mening* OR myeli* OR myalg* OR ADEM OR ataxi* OR dysphasi* OR aphasi* OR stroke OR guillain-barre OR Miller-Fisher OR paresis OR palsy OR cerebr* OR crani* OR epilep* OR seizure or headache* OR migraine* OR demyeli* OR neuroimag* OR neurotrop* OR neuroinvas* OR neuropath* OR cerebrospinal* or cerebro-spinal OR CSF OR*deliri* OR sleep OR insomnia OR somnolence OR hypersomnolence OR parasomnia OR "movement disorder" OR neuropsych* OR dement* OR cogniti* OR irritability OR hallucinat* OR delusion* OR apath* OR indifference OR agitat* OR euphori* OR elation OR elated OR disinhibit* OR aggressi* OR amnes* OR catatoni* OR personality OR psycho* OR mental OR mood OR affective OR depress* OR anxi* OR "obsessive compulsive" OR OCD OR "panic disorder" OR post-trauma* OR posttrauma* OR PTSD OR neurosis OR neurotic OR bipolar OR mania OR manic OR schizophreni* OR "intelligence quotient" OR IQ OR "mental retardation" OR "intellectual disability" OR "learning disability" OR autis* OR asperger* OR "attention deficit" OR ADHD OR hyperactivity OR hyperkinetic OR suicid* OR emotion* OR appetite OR fatigu* OR tired* OR confus*).ti,ab OR

(exp Psychiatry/ OR exp Sensory System Disorders/ OR exp Sense Organ Disorders/ OR exp Nervous System Disorders/ OR exp Neurosciences/ or exp Emotional States/ OR exp DELIRIUM/ OR exp "NEUROCOGNITIVE DISORDERS"/ OR exp "SLEEP WAKE DISORDERS"/ OR exp INSOMNIA/ OR exp HYPERSOMNIA/ OR exp "MOVEMENT DISORDERS"/ OR exp DEMENTIA/ OR exp "COGNITIVE ABILITY"/ OR exp "COGNITIVE IMPAIRMENT"/ OR exp "NEUROCOGNITIVE DISORDERS"/ OR exp IRRITABILITY/ OR exp HALLUCINATIONS/ OR exp DELUSIONS/ OR exp APATHY/ OR exp AGITATION/ OR exp EUPHORIA/ OR exp "BEHAVIORAL DISINHIBITION"/ OR exp AMNESIA/ OR exp CATATONIA/ OR exp PERSONALITY/ OR exp "PERSONALITY DISORDERS"/ OR exp PSYCHOSIS/ OR exp "MENTAL DISORDERS"/ OR exp EMOTIONS/ OR exp "AFFECTIVE DISORDERS"/ OR exp "DEPRESSION (EMOTION)"/ OR exp ANXIETY/ OR exp "ANXIETY DISORDERS"/ OR exp "OBSESSIVE COMPULSIVE DISORDER"/ OR exp "PANIC DISORDER"/ OR exp "POSTTRAUMATIC STRESS"/ OR exp NEUROSIS/ OR exp "BIPOLAR DISORDER"/ OR exp MANIA/ OR exp SCHIZOPHRENIA/ OR exp "INTELLIGENCE QUOTIENT"/ OR exp "NEURODEVELOPMENTAL DISORDERS"/ OR exp "INTELLECTUAL DEVELOPMENT DISORDER"/ OR exp "AUTISM SPECTRUM DISORDERS"/ OR exp "ATTENTION DEFICIT DISORDER"/ OR exp HYPERKINESIS/ OR exp SUICIDE/ OR exp EMOTIONS/ OR exp APPETITE/ OR exp "EATING DISORDERS"/ OR exp FATIGUE/ OR exp "MENTAL CONFUSION"/)

[Population Human]

**MedRxiv**

(Monkeypox OR monkeypox OR monkey pox OR MPV)

In categories psychiatry and clinical psychology; neurology
