# Supplementary material for: Neurological and psychiatric presentations associated with human monkeypox virus infection: A systematic review and meta-analysis
Source: eClinicalMedicine. 2022 Sep 8;52:101644. doi: 10.1016/j.eclinm.2022.101644 (PMC9533950; doi:10.1016/j.eclinm.2022.101644)
Supplement: Supplementary file 8 [file mmc8.docx]

**Supplementary Table 2 Reasons for excluding studies at full text screening**

| **Title** | **Year** | **Journal** | **Volume** | **Issue** | **Page** | **Authors** | **Reason for exclusion** | |
| --- | --- | --- | --- | --- | --- | --- | --- | --- |
| Common clinical complications of human monkeypox infection at the general hospital of kole, in Democratic Republic of Congo | 2014 | American Journal of Tropical Medicine and Hygiene | 91 | 5 | 139 | Mbala P.K. and Martin J.W. and Huggins J.W. and Muyembe J.J. and Mutambay C.K. and Soltis B. and Rimoin A.W. and Guerena F.B. and Korman L. and Pitman P. | Duplicate | |
| Family cluster of three cases of monkeypox imported from Nigeria to the United Kingdom, May 2021 | 2021 | Eurosurveillance | 26 | 32 |  | Hobson G. and Adamson J. and Adler H. and Firth R. and Gould S. and Houlihan C. and Johnson C. and Porter D. and Rampling T. and Ratcliffe L. and Russell K. and Shankar A.G. and Wingfield T. | No neuro/psych symptoms | |
| Monitoring healthcare professionals after monkeypox exposure: Experience from the first case imported to Asia | 2020 | Infection Control and Hospital Epidemiology | 41 | 3 | 373-375 | Kyaw W.M. and Vasoo S. and Ho H.J.A. and Chan M. and Yeo T.W. and Manauis C.M. and Ang H. and Pratim De P. and Ang B.S.P. and Chow A.L.P. | No neuro/psych symptoms | |
| Human monkeypox in Sierra Leone after 44-year absence of reported cases | 2019 | Emerging Infectious Diseases | 25 | 5 | 1023-1025 | Reynolds M.G. and Wauquier N. and Li Y. and Satheshkumar P.S. and Kanneh L.D. and Monroe B. and Maikere J. and Saffa G. and Gonzalez J.-P. and Fair J. and Carroll D.S. and Jambai A. and Dafae F. and Khan S.H. and Moses L.M. | No neuro/psych symptoms | |
| Presumptive risk factors for monkeypox in rural communities in the Democratic Republic of the Congo | 2017 | PLoS ONE | 12 | 2 | e0168664 | Quiner C.A. and Moses C. and Monroe B.P. and Nakazawa Y. and Doty J.B. and Hughes C.M. and Mccollum A.M. and Ibata S. and Malekani J. and Okitolonda E. and Carroll D.S. and Reynolds M.G. | No neuro/psych symptoms | |
| Monkeypox detection in maculopapular lesions in two young Pygmies in the Central African Republic | 2012 | International Journal of Infectious Diseases | 16 |  | e266-e267 | Nakoune E. and Kazanji M. | No neuro/psych symptoms | |
| Human monkeypox: confusion with chickenpox. | 1988 | Acta tropica | 45 | 4 | 297-307 | Jezek, Z and Szczeniowski, M and Paluku, K M and Mutombo, M and Grab, B | No neuro/psych symptoms | |
| Temporal and Spatial Dynamics of Monkeypox in Democratic Republic of Congo, 2000-2015 | 2019 | EcoHealth | 16 | 3 | 476-487 | Mandja B.-A.M. and Brembilla A. and Handschumacher P. and Bompangue D. and Gonzalez J.-P. and Muyembe J.-J. and Mauny F. AO - Mandja, Bien-Aime Makasa; ORCID: https://orcid.org/0000-0003-3846-5746 | No neuro/psych symptoms | |
| Detection of monkeypox virus with real-time PCR assays | 2006 | Journal of Clinical Virology | 36 | 3 | 194-203 | Li Y. and Olson V.A. and Laue T. and Laker M.T. and Damon I.K. | No neuro/psych symptoms | |
| Do Monkeypox Exposures Vary by Ethnicity? Comparison of Aka and Bantu Suspected Monkeypox Cases. | 2020 | The American journal of tropical medicine and hygiene | 102 | 1 | 202-205 | Guagliardo, Sarah Anne J and Doshi, Reena H and Reynolds, Mary G and Dzabatou-Babeaux, Angelie and Ndakala, Nestor and Moses, Cynthia and McCollum, Andrea M and Petersen, Brett W | No neuro/psych symptoms | |
| Confidence in managing human monkeypox cases in Asia: A cross-sectional survey among general practitioners in Indonesia | 2020 | Acta Tropica | 206 |  | 105450 | Harapan H. and Setiawan A.M. and Yufika A. and Anwar S. and Wahyuni S. and Asrizal F.W. and Sufri M.R. and Putra R.P. and Wijayanti N.P. and Salwiyadi S. and Khusna A. and Nusrina I. and Shidiq M. and Fitriani D. and Muharrir M. and Husna C.A. and Yusri F. and Maulana R. and Utomo P.S. and Andalas M. and Wagner A.L. and Mudatsir M. | Not infected with MPV | |
| Knowledge of human monkeypox viral infection among general practitioners: a cross-sectional study in Indonesia | 2020 | Pathogens and Global Health | 114 | 2 | 68-75 | Harapan H. and Setiawan A.M. and Yufika A. and Anwar S. and Wahyuni S. and Asrizal F.W. and Sufri M.R. and Putra R.P. and Wijayanti N.P. and Salwiyadi S. and Khusna A. and Nusrina I. and Shidiq M. and Fitriani D. and Muharrir M. and Husna C.A. and Yusri F. and Maulana R. and Andalas M. and Wagner A.L. and Mudatsir M. AO - Harapan, Harapan; ORCID: http://orcid.org/0000-0001-7630-8413 AO - Mudatsir, Mudatsir; ORCID: http://orcid.org/0000-0002-5643-9384 AO - Anwar, Samsul; ORCID: http://orcid.org/0000-0003-3165-2151 AO - Nusrina, Ina; ORCID: http://orcid.org/0000-0003-4000-1123 AO - Wagner, Abram L.; ORCID: http://orcid.org/0000-0003-4691-7802 | Not infected with MPV | |
| Enhancing health care worker ability to detect and care for patients with monkeypox in the democratic republic of the Congo | 2013 | International Health | 5 | 4 | 237-243 | Bass J. and Tack D.M. and McCollum A.M. and Kabamba J. and Pakuta E. and Malekani J. and Nguete B. and Monroe B.P. and Doty J.B. and Karhemere S. and Damon I.K. and Balilo M. and Okitolonda E. and Shongo R.L. and Reynolds M.G. | Not infected with MPV | |
| The changing epidemiology of human monkeypox - a potential threat? A systematic review | 2021 | medRxiv |  |  |  | Bunge E.M. and Hoet B. and Chen L. and Lienert F. and Weidenthaler H. and Baer L.R. and Steffen R. AO - Bunge, Eveline M.; ORCID: https://orcid.org/0000-0001-7988-909X AO - Hoet, Bernard; ORCID: https://orcid.org/0000-0002-5345-3236 AO - Lienert, Florian; ORCID: https://orcid.org/0000-0002-9161-7107 AO - Chen, Liddy; ORCID: https://orcid.org/0000-0001-6569-9674 AO - Weidenthaler, Heinz; ORCID: https://orcid.org/0000-0002-6866-4028 AO - Baer, Lorraine R.; ORCID: https://orcid.org/0000-0002-2070-8503 AO - Steffen, Robert; ORCID: https://orcid.org/0000-0001-7917-1141 | Not original data | |
| Re-Emergence of monkeypox amidst delta variant concerns: A point of contention for public health virology? | 2022 | Journal of Medical Virology | 94 | 3 | 805-806 | Sarwar S. and Maskey U. and Thada P.K. and Mustansir M. and Sarfraz A. and Sarfraz Z. AO - Sarfraz, Azza; ORCID: https://orcid.org/0000-0001-8206-5745 AO - Sarfraz, Zouina; ORCID: https://orcid.org/0000-0002-5132-7455 | Not original data | |
| Monkeypox contacts: a puzzling problem | 2018 | The Lancet | 392 | 10152 | 986 | The Lancet | Not original data | |
| Physicians' willingness to be vaccinated with a smallpox vaccine to prevent monkeypox viral infection: A cross-sectional study in Indonesia | 2020 | Clinical Epidemiology and Global Health | 8 | 4 | 1259-1263 | Harapan H. and Setiawan A.M. and Yufika A. and Anwar S. and Wahyuni S. and Asrizal F.W. and Sufri M.R. and Putra R.P. and Wijayanti N.P. and Salwiyadi S. and Khusna A. and Nusrina I. and Shidiq M. and Fitriani D. and Muharrir M. and Husna C.A. and Yusri F. and Maulana R. and Itoh N. and Andalas M. and Wagner A.L. and Mudatsir M. AO - Harapan, Harapan; ORCID: http://orcid.org/0000-0001-7630-8413 AO - Mudatsir, Mudatsir; ORCID: http://orcid.org/0000-0002-5643-9384 AO - Setiawan, Abdul M.; ORCID: http://orcid.org/0000-0002-6913-2007 AO - Anwar, Samsul; ORCID: http://orcid.org/0000-0003-3165-2151 AO - Nusrina, Ina; ORCID: http://orcid.org/0000-0003-4000-1123 AO - Wagner, Abram L.; ORCID: http://orcid.org/0000-0003-4691-7802 | Not original data | |
| Emerging uk infections: Identifying new threats early | 2019 | Prescriber | 30 | 3 | 21-24 | Greener M. | Not original data | |
| The evolving global epidemiology of encephalitis | 2013 | Tropical Medicine and International Health | 18 |  | 28 | Sejvar J. | Not original data | |
| The global burden of bacterial and viral zoonotic infections | 2011 | Clinical Microbiology and Infection | 17 | 3 | 326-330 | Christou L. | Not original data | |
| Human monkeypox: An emerging zoonotic disease | 2007 | Future Microbiology | 2 | 1 | 17-34 | Parker S. and Nuara A. and Buller R.M.L. and Schultz D.A. | Not original data | |
| Emerging infections and pregnancy: West nile virus, monkeypox, severe acute respiratory syndrome, and bioterrorism | 2005 | Clinics in Perinatology | 32 | 3 | 765-776 | Jamieson D.J. and Jernigan D.B. and Ellis J.E. and Treadwell T.A. | Not original data | |
| The evolving epidemiology of viral encephalitis | 2006 | Current Opinion in Neurology | 19 | 4 | 350-357 | Sejvar J.J. | Not original data | |
| Zoonotic viral diseases and the frontier of early diagnosis, control and prevention | 2006 | Journal of Internal Medicine | 260 | 5 | 399-408 | Heeney J.L. | Not original data | |
| SARS: Here to stay? Monkeypox: Beware of exotic pets | 2003 | Cleveland Clinic Journal of Medicine | 70 | 10 | 889-895 | Gordon S.M. and Longworth D.L. | Not original data | |
| Aetiological study of viruses causing acute encephalitis syndrome in North West India. | 2017 | Indian journal of medical microbiology | 35 | 4 | 529-534 | Tiwari, Jitendra Kumar and Malhotra, Bharti and Chauhan, Aradhana and Malhotra, Hemant and Sharma, Pratibha and Deeba, Farah and Trivedi, Khushbu and Swamy, Anjenya M | Not original data | |
| Reemergence of monkeypox: prevalence, diagnostics, and countermeasures. | 2005 | Clinical infectious diseases : an official publication of the Infectious Diseases Society of America | 41 | 12 | 1765-71 | Nalca, Aysegul and Rimoin, Anne W and Bavari, Sina and Whitehouse, Chris A | Not original data | |
| 21st-century emerging and reemerging infections. | 2004 | The American journal of nursing | 104 | 1 | 67-70 | Goldrick, Barbara A | Not original data | |
| Public health implications of emerging zoonoses. | 2000 | Revue scientifique et technique (International Office of Epizootics) | 19 | 1 | 310-7 | Meslin, F X and Stohr, K and Heymann, D | Not original data | |
| Systematic review of important viral diseases in africa in light of the 'one health' concept | 2020 | Pathogens | 9 | 4 | 301 | Chauhan R.P. and Dessie Z.G. and Noreddin A. and El Zowalaty M.E. AO - Chauhan, Ravendra P.; ORCID: http://orcid.org/0000-0002-4674-8255 AO - Dessie, Zelalem G.; ORCID: http://orcid.org/0000-0001-9056-6822 AO - Noreddin, Ayman; ORCID: http://orcid.org/0000-0003-4899-1168 AO - El Zowalaty, Mohamed E.; ORCID: http://orcid.org/0000-0002-1056-4761 | Not original data | |
| A systematic review of the epidemiology of human monkeypox outbreaks and implications for outbreak strategy | 2019 | PLoS Neglected Tropical Diseases | 13 | 10 | e0007791 | Beer E.M. and Bhargavi Rao V. | Not original data | |
| Improving the care and treatment of monkeypox patients in low-resource settings: Applying evidence from contemporary biomedical and smallpox biodefense research | 2017 | Viruses | 9 | 12 | 380 | Reynolds M.G. and McCollum A.M. and Nguete B. and Lushima R.S. and Petersen B.W. | Not original data | |
| Viral infections of the face | 2014 | Clinics in Dermatology | 32 | 6 | 715-733 | Avci O. and Ertam I. | Not original data | |
| Comparative Pathology of Smallpox and Monkeypox in Man and Macaques | 2013 | Journal of Comparative Pathology | 148 | 1 | Jun-21 | Cann J.A. and Jahrling P.B. and Hensley L.E. and Wahl-Jensen V. | Not original data | |
| Human smallpox (monkeypox, cowpox and Yatapox) | 2011 | DoctorConsult - The Journal. Wissen fur Klinik und Praxis | 2 | 4 | e229-e234 | Gurtler L. | Not original data | |
| Zoonoses associated with petting farms and open zoos | 2008 | Vector-Borne and Zoonotic Diseases | 8 | 1 | 85-92 | Stirling J. and Griffith M. and Dooley J.S.G. and Goldsmith C.E. and Loughrey A. and Lowery C.J. and McClurg R. and McCorry K. and McDowell D. and McMahon A. and Millar B.C. and Rao J. and Rooney P.J. and Snelling W.J. and Matsuda M. and Moore J.E. | Not original data | |
| Orthopoxvirus: Biology, pathology and therapy | 2008 | Drugs of the Future | 33 | 10 | 875-890 | Byrd C.M. and Page J. and Hruby D.E. and Jordan R. | Not original data | |
| Fever of Unknown Origin Due to Zoonoses | 2007 | Infectious Disease Clinics of North America | 21 | 4 | 963-996 | Cleri D.J. and Ricketti A.J. and Vernaleo J.R. | Not original data | |
| Reasons for the increase in emerging and re-emerging viral infectious diseases | 2006 | Microbes and Infection | 8 | 3 | 905-916 | Ka-Wai Hui E. | Not original data | |
| Emerging infectious diseases at the beginning of the 21st century | 2006 | Online journal of issues in nursing | 11 | 1 | 2 | Lashley F.R. | Not original data | |
| Globalization and infectious diseases | 2006 | Recenti Progressi in Medicina | 97 | 10 | 528-532 | Garavelli P.L. and Peduzzi P. | Not original data | |
| Overview and summary: Infectious diseases: Challenges and solutions | 2006 | Online Journal of Issues in Nursing | 11 | 1 |  | Couig M.P. and Admiral R. | Not original data | |
| Emerging zoonotic epidemics in the interconnected global community | 2005 | Veterinary Record | 157 | 22 | 673-679 | Gibbs E.P.J. | Not original data | |
| Life-threatening cutaneous viral diseases | 2005 | Clinics in Dermatology | 23 | 2 | 157-163 | Rebora A. | Not original data | |
| New and emerging infectious diseases | 2005 | Journal of the American Academy of Dermatology | 52 | 6 | 1062-1068 | Elston D.M. | Not orginal data | |
| Triage of a febrile patient with a rash: A comparison of chickenpox, monkeypox, and smallpox | 2004 | Disaster Management and Response | 2 | 3 | 81-86 | Not orginal data | |  |
| Emerging infectious disease: Vulnerabilities, contributing factors and approaches | 2004 | Expert Review of Anti-Infective Therapy | 2 | 2 | 299-316 | Lashley F.R. | Not orginal data | |
| Cutaneous manifestations of viral infections | 2004 | Hong Kong Practitioner | 26 | 1 | 31-41 | Not orginal data | |  |
| Monkey-pox, a model of emergent then reemergent disease | 2004 | Medecine et Maladies Infectieuses | 34 | 1 | Dec-19 | Georges A.-J. and Matton T. and Courbot-Georges M.-C. | Not orginal data | |
| Anticipating smallpox and monkeypox outbreaks: Complications of the smallpox vaccine | 2004 | Neurologist | 10 | 5 | 265-274 | Abrahams B.C. and Kaufman D.M. | Not orginal data | |
| WHO consults on the challenges of predicting zoonotic disease | 2004 | Veterinary Record | 154 | 20 | 611-612 | Anonymous. | Not orginal data | |
| Exotic pets and monkeypox | 2003 | Infections in Medicine | 20 | 9 | 410 | Estrada B. | Not orginal data | |
| Risks and prevention of nosocomial transmission of rare zoonotic diseases | 2001 | Clinical Infectious Diseases | 32 | 3 | 446-456 | Weber D.J. and Rutala W.A. | Not orginal data | |
| The smallpox story: Life and death of an old disease | 1983 | Microbiological Reviews | 47 | 4 | 455-509 | Not orginal data | |  |
| Suicide, self-harm and thoughts of suicide or self-harm in infectious disease epidemics: a systematic review and meta-analysis. | 2021 | Epidemiology and psychiatric sciences | 30 | 101561091 | e32 | Not orginal data | |  |
| Pathogen-host associations and predicted range shifts of human monkeypox in response to climate change in central Africa. | 2013 | PloS one | 8 | 7 | e66071 | Thomassen, Henri A and Fuller, Trevon and Asefi-Najafabady, Salvi and Shiplacoff, Julia A G and Mulembakani, Prime M and Blumberg, Seth and Johnston, Sara C and Kisalu, Neville K and Kinkela, Timothee L and Fair, Joseph N and Wolfe, Nathan D and Shongo, Robert L and LeBreton, Matthew and Meyer, Hermann and Wright, Linda L and Muyembe, Jean-Jacques and Buermann, Wolfgang and Okitolonda, Emile and Hensley, Lisa E and Lloyd-Smith, James O and Smith, Thomas B and Rimoin, Anne W | Not orginal data | |
| [Emerging viral diseases]. | 2006 | Infections virales emergentes. | 190 | 3 | 597-7 | Not original data | |  |
| [Zoonoses in children from new pets]. | 2005 | Zoonoses chez l'enfant et nouveaux animaux de compagnie. | 35 | 311416 | S117-20 | Quinet, B | Not orginal data | |
| Tropical dermatology: viral tropical diseases. | 2003 | Journal of the American Academy of Dermatology | 49 | 6 | 979-2 | Not orginal data | |  |
| Smallpox vaccination and bioterrorism with pox viruses. | 2003 | Comparative immunology, microbiology and infectious diseases | 26 | 5 | 423-30 | Mayr, Anton | Not orginal data | |
| Emerging viral infections of the central nervous system: Part 1. | 2009 | Archives of Neurology | 66 | 8 | 939-948 | Not orginal data | |  |
| Comparative proteomics of human monkeypox and vaccinia intracellular mature and extracellular enveloped virions | 2008 | Journal of Proteome Research | 7 | 3 | 960-968 | Manes N.P. and Estep R.D. and Mottaz H.M. and Moore R.J. and Clauss T.R.W. and Monroe M.E. and Du X. and Adkins J.N. and Wong S.W. and Smith R.D. | Pre-clinical/ purely mechanistic data | |
| Risk factors associated with human monkeypox in the democratic republic of Congo | 2014 | American Journal of Tropical Medicine and Hygiene | 91 | 5 | 199-200 | Hoff N. and Mulembakani P.M. and Johnston S.C. and Kisalu N.K. and Muyembe J.-J. and Hensley L.E. and Okitolonda E. and Rimoin A.W. | Unable to access article/ full text | |
| Complications of monkeypox infections in humans | 2011 | American Journal of Tropical Medicine and Hygiene | 85 | 6 | 396 | Mbala P.K. and Huggins J. and Muyembe J.-J. and Cesar C.K. and Withers M. and Soltis B. and Martin J. and Guerena F.B. and Pittman P.R. | Unable to access article/ full text | |
| Clinical observations of human monkeypox infections in the democratic republic of the Congo | 2011 | American Journal of Tropical Medicine and Hygiene | 85 | 6 | 393 | Martin J. and Withers M. and Huggins J. and Muyembe J.-J. and Placide M.-K. and Cesar C.L. and Soltis B. and Guerena F.B. and Korman L. and Pittman P.R. | Unable to access article/ full text | |
| Towards enhanced surveillance for monkeypox: Application of a robust clinical case definition | 2010 | American Journal of Tropical Medicine and Hygiene | 83 | 5 | 122 | Unable to access article/ full text | |  |
| Monkeypox confirmed in the USA | 2003 | Veterinary Record | 152 | 25 | 759 | Anonymous. | Unable to access article/ full text | |
| Public Health England Annual Conference 2019 | 2019 | The Lancet Public Health | 4 | 10 | e497 | Carson J. | Unable to access article/ full text | |
| Ocular complications associated with acute monkeypox virus infection, DRC | 2014 | International Journal of Infectious Diseases | 21 |  | 276-277 | Hughes C. and McCollum A. and Pukuta E. and Karhemere S. and Nguete B. and Shongo Lushima R. and Kabamba J. and Balilo M. and Muyembe Tamfum J.-J. and Wemakoy O. and Malekani J. and Monroe B. and Damon I. and Reynolds M. | Unable to access article/ full text | |
| The relationship between neuroinflammation, MPV and Parkinson's disease | 2013 | European Geriatric Medicine | 4 | 6 | 418-419 |  | Unable to access article/ full text | |
| Monkeypox Pathogenesis Study Using A Serial Sacrifice Technique | 2010 | American Journal of Tropical Medicine and Hygiene | 83 | 5 | 347-348 |  | Unable to access article/ full text | |
| Superinfections create further HIV vaccine complications | 2003 | Expert Review of Anti-Infective Therapy | 1 | 2 | 199-203 | Anonymous. | Unable to access article/ full text | |
| The clinical characterization of human monkeypox infections in the Democratic Republic of Congo | 2017 | Tropical Medicine and International Health | 22 |  | 31 |  | Unable to access article/ full text | |
