# Supplementary material for: Neurological and psychiatric presentations associated with human monkeypox virus infection: A systematic review and meta-analysis
Source: eClinicalMedicine. 2022 Sep 8;52:101644. doi: 10.1016/j.eclinm.2022.101644 (PMC9533950; doi:10.1016/j.eclinm.2022.101644)
Supplement: Supplementary file 9 [file mmc9.docx]

Supplementary Table 3 Freeman-Tukey sensitivity meta-analysis

| **Symptom** | **N studies** | **N subjects** | **Pooled prevalence** | **95% CI** | **I^2^** |
| --- | --- | --- | --- | --- | --- |
| Seizures | 2 | 74 | 0.0269 | [0.0000, 0.0825] | 0.0% |
| Confusion | 2 | 250 | 0.0255 | [0.0001, 0.0777] | 46.2% |
| Encephalitis | 3 | 283 | 0.0232 | [0.0000, 0.0857] | 68.9% |
